# Increased phenotypic diversity as a consequence of ecological opportunity in the island radiation of Sulawesi ricefishes (Teleostei: Adrianichthyidae)

**DOI:** 10.1186/s12862-025-02355-1

**Published:** 2025-03-12

**Authors:** Jan Möhring, Sebastian Hüllen, Sebastian Martin, Daniel F. Mokodongan, Daisy Wowor, Julia Schwarzer, Fabian Herder

**Affiliations:** 1 Museum Koenig Bonn, Leibniz Institute for the Analysis of Biodiversity Change, Adenauerallee 127, D- 53113 Bonn, Germany; 2 Research Center for Biosystematics and Evolution, Museum Zoologicum Bogoriense, National Research and Innovation Agency (BRIN), Jalan Raya Bogor Km 46, Cibinong, 16911 Indonesia

**Keywords:** Adrianichthyidae, Ecological opportunity, Adaptive diversification, Sulawesi, Geometric morphometrics, Phenotype evolution, Evolutionary radiation, Wallacea

## Abstract

Ecological opportunity is considered a main driver of adaptive diversification. Colonization of new areas and habitats provides novel opportunities, which may lead to phenotypic change and rapid diversification. The geographically isolated island Sulawesi in Indonesia hosts numerous endemic radiations. The ricefish family Adrianichthyidae is the most speciose of the few exclusively freshwater-dwelling fish lineages that colonized the tropical island. While the family’s distribution includes large parts of Asia, the monophyletic lineage endemic to Sulawesi comprises more than half of all species described, with conspicuous morphological diversity. Using shape and size as proxies for phenotypic and ecological diversity, we first test the hypothesis that Sulawesi ricefishes are more diverse than non-Sulawesi species. We then ask if adaptation to alternative macrohabitats, namely rivers and lakes, can explain phenotypic diversification. We find that Sulawesi ricefishes exhibit greater disparity in body and head shape than non-Sulawesi ricefishes. By including phylogenomic data, we demonstrate that shape diversity in Sulawesi evolved along multiple trajectories, with reduced phylogenetic influence. Changes in body depth and head elongation are prominent, indicating distinct ecological drivers. In particular, body depth evolves along the river-lake axis, and lake species are more diverse compared to riverine ricefishes. In some lacustrine species, pronounced changes in head shape, a trait commonly associated with feeding in fishes, suggest trophic adaptation. Rates of phenotype evolution are higher in the Sulawesi lineages, in particular in lacustrine species. Lastly, we identified opposing trends in size evolution, with generally larger bodies in Sulawesi and repeated miniaturization in non-Sulawesi species. Our results indicate that ecological opportunity greatly influenced the observed phenotype diversity. Low diversity of freshwater fish lineages created environments with reduced competition, whereas additional factors, such as the island’s complex geological history, created diverse habitats. As reflected in very localized endemism, species diversity is possibly also driven by long-term spatial isolation, which alone however offers insufficient explanation for the increase in phenotypic disparity. Taken together, ecological opportunity was a dominant driver of the phenotypic diversification of Sulawesi ricefishes, potentially complemented by past introgressive hybridization events and small-scale spatial isolation.

**Supplementary Information:**

The online version contains supplementary material available at 10.1186/s12862-025-02355-1.

## Introduction

Ecological opportunity, a term describing available ecological resources that populations encounter, is considered a key driver of evolution, and can promote rapid speciation and morphological diversification [1–4]. Among the processes leading to ecological opportunity, colonization of new areas is considered the most well-known. Isolated ecosystems can provide conditions to invading organisms, which are unavailable to them in their original range. These conditions may include the absence of competing or predatory lineages, but access to novel resources can also be facilitated by the evolution of key innovations [2, 4–6]. These conditions of ecological release commonly result in character displacement, the adaptive diversification of functional traits [2–4, 6, 7]. Adaptations to specific modes of resource exploitation foster adaptive diversification, and promoted island and lake radiations across the tree of life [8].

**Fig. 1:**
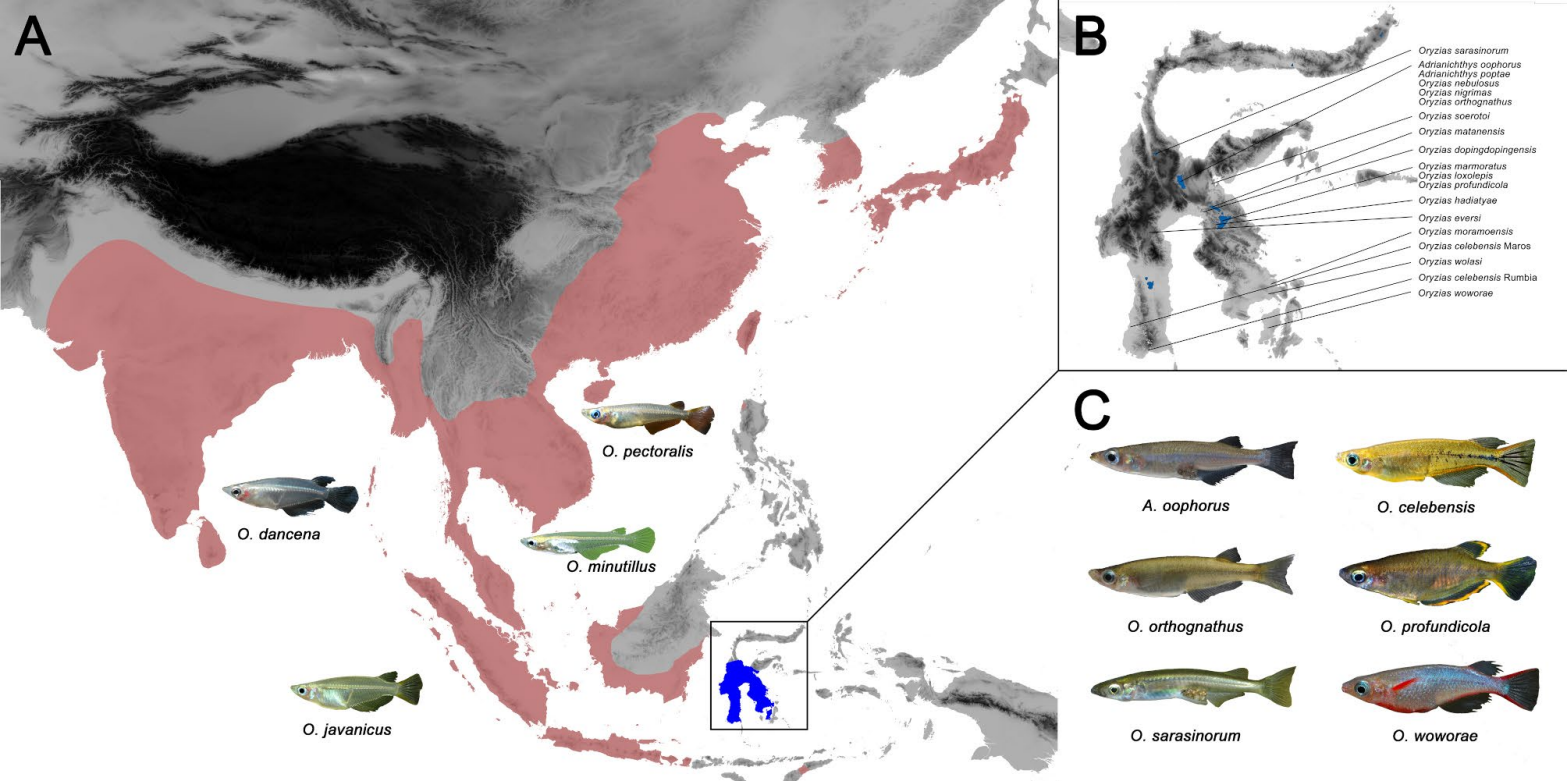
Overview of the distribution of ricefishes and representative species. (**A**) The ricefish family is widespread across southern, southeastern and eastern Asia (red: approximate distribution outside of Sulawesi, blue: approximate distribution in Sulawesi). (**B**) Although only representing a tiny fraction of the distribution, the Indonesian island of Sulawesi harbors an unequal portion of the taxonomic diversity, with more than half of all known species. All of these species are locally endemic on the island. (**C**) Phenotypic diversity also appears to be more diverse in Sulawesi, suggesting evolutionary drivers promoting diversification. Pictures with kind permission by H.-G. Evers, A. Wagnitz, F. Schäfer and J. Flury

Ricefishes (Adrianichthyidae) are a family of small teleost fishes belonging to the order Beloniformes, which also includes needlefish, halfbeaks and flying fish [9]. They occur in fresh- and brackish waters of South, East and South-East Asia, where they inhabit mostly stagnant or slow-flowing waters (including the eponymous rice paddies), swamps, brackish water mangroves and lagoons. Currently, 41 species are described throughout their distribution range [9, 10]. Selected species, especially *O. latipes*, are of great importance as model organisms, e.g. in developmental biology, genetics and toxicology, as well as in ornamental fish keeping [11]. At present, two genera, *Oryzias* and *Adrianichthys*, are recognized, although the former genus has been unanimously shown to be paraphyletic in phylogenomic studies, and proposals for a generic revision of the family have already been made [12]. Despite representing only a small part of the family’s distribution area, 24 species, more than half of all described ricefish species, are endemic to Sulawesi, the largest island of the Wallacea region in the Indonesian archipelago [10, 13] (Fig. 1). From a biogeographic perspective, Sulawesi is unique due to its continuous isolation from the continental shelf while remaining in its close proximity. This allowed for biotic assembly through a plethora of dispersal events [14, 15], giving rise to a variety of independent organismic radiations [16–21]. Only a limited number of freshwater teleost lineages dispersed across the marine barriers and colonized the island [15, 22, 23], and primary freshwater fishes like cypriniforms are naturally absent. Instead, Sulawesi freshwaters are dominated by lineages of ancestral marine origin, with diadromous life cycles, or higher osmotic tolerance [24, 25].

Multiple phylogenetic studies have shown that Sulawesi ricefishes are monophyletic (e.g [12, 26–30]), and most likely date back to one single colonization event [13, 26, 27, 29, 31], with estimates ranging from 4 to 47 Mya [12, 15, 30, 32]. While phylogenetic relationships among ricefish species are generally well understood, phenotypic diversity and its evolution have not been studied in detail so far. Superficially, Sulawesi ricefishes appear to be remarkably diverse in terms of external characteristics such as body size and body and head shapes compared to other ricefishes, e.g [9, 13]. One example are the species of the genus *Adrianichthys*, which are endemic to Lake Poso and differ morphologically from the other ricefish to such a degree, that they were previously classified in a family of their own [9, 33, 34]. However, it has repeatedly been shown that they are nested within *Oryzias* in molecular phylogenetic and phylogenomic studies (e.g [12, 26–30]).

In fishes, ecological opportunity is tightly linked to the availability of specific habitats and food resources. Greater ecological opportunity in lakes has been proposed as an explanation for high rates of diversification of fishes in lacustrine environments compared to those in rivers [35]. Ricefishes have colonized most areas of Sulawesi, except for the northern peninsula [9, 12, 25], and inhabit both rivers and lakes, including the island’s large ancient lakes. Out of the currently described 24 Sulawesi ricefish species, seven are riverine, while the remaining ones are lacustrine. Here, we present the first comprehensive analysis of quantitative phenotypic variation across the ricefish family. We use geometric morphometric shape data and standard lengths to explore axes of trait diversity, and test whether the combined trait ranges and morphospace occupation of Sulawesi ricefish species exceed those of non-Sulawesi species, and which trajectories of trait change are responsible for the observed phenotypic disparity.

We hypothesize further that the Sulawesi radiation of ricefishes adapted along alternative trajectories to lake vs. river conditions. We consider three possible scenarios of shape evolution in response to habitat: (0) In case of the null hypothesis, we expect no significant morphospace partitioning between lacustrine and riverine forms. (1) In case of selection on specific phenotypes of riverine ancestors in lacustrine environments, we expect to see a selection of shape states shared with riverine taxa. This lack of differentiated diversification would also disagree with ecological opportunity as a driver. (2) Second, we considered directional displacement towards a shared or ‘typical’ lacustrine shape phenotype following a common trajectory in lakes, resulting in partial or no morphospace overlap with the riverine species set. For example, similar character displacements in form of increases in body depth have repeatedly been shown in fish transitioning from lotic to lentic waters [36–38], including ricefish [31]. (3) Lastly, we considered a generally greater shape diversity for the lacustrine group with clear character displacement beyond the boundaries of other groups following multiple independent trajectories, suggesting different drivers of diversification. This pattern would fit an ecological opportunity scenario best, since character displacement is expected to promote phenotypic divergence into novel phenotypes, filling previously unoccupied areas of morphospace [2–4]. In addition to morphospace occupation, we hypothesize that phylogenetic signal is lower in the Sulawesi group, while rates of shape evolution are enhanced.

To evaluate these hypotheses, we compared body and head shapes, as well as standard lengths, of 24 described ricefish species from the Sulawesi radiation and additional species from outside the island using quantitative landmark data obtained from skeletal features. For each group, as well as for each sex, we explore axes of trait diversity and contrast morphospace occupation as a proxy for phenotypic evolution with a whole-genome based molecular phylogeny. Similarly, we compare lacustrine and riverine species sets to investigate macrohabitat as a main driver of trait diversification. Finally, we reconstruct ancestral states to compare ancestral forms and trends of phenotypic evolution after colonization of Sulawesi.

## Materials and methods

### Specimen selection, taxonomic approach and X-ray acquisition

The present study is based on the analysis of museum material of Sulawesi ricefishes, most of which was collected during fieldwork between 2002 and 2013, covering 17 of the 24 species formally recognized from the island at present (Suppl. Tab. S1) [10]. Ricefishes from outside Sulawesi, included for comparative analyses on shape diversity patterns of the Sulawesi ricefishes, are covered by a total of seven out of 17 described *Oryzias* species. Genetically clearly divergent lineages from different geographic locations, which are collectively referred to as one species at present, were treated as distinct OTUs (Operational Taxonomic Unit). This was relevant for *Oryzias celebensis* from different river basins in South-West Sulawesi (see e.g [12, 26, 31]). For each of these species, genetically divergent lineages from outside the type locality were treated as distinct OTUs. For one population of *O. celebensis* (‘Rumbia’), no genomic data was available for specimens originating from the corresponding Kelara River drainage. However, we linked them with genomic data of the geographically proximate ‘Malino’ population from the same mountain range. One *Oryzias* specimen from Lake Towuti could not be identified using the key provided by [39], as characters were ambiguous, and was eventually assigned to *O. loxolepis* based on its general appearance. Four species from Sulawesi, the enigmatic and possibly extinct *Adrianichthys kruyti* and *A. roseni* from Lake Poso, *O. bonneorum* from Lake Lindu, and the recently described *O. kalimpaaensis*, could not be included here due to a lack of available or suitable specimens. Two species, *O. asinua* from Sulawesi and *O. uwai* from mainland Asia, had to be removed from the dataset because they were only represented by a single suitable specimen.

Species identification followed the recent taxonomic literature [9, 28, 39–46]. Two to 55 (median: 14; mean 18.6) specimens per species were analyzed, depending on availability (see Suppl. Table S1).

We selected mature specimens without visible deformations, bending or external damage affecting shape. Males and females were distinguished based on the presence or absence of elongated dorsal and anal fin rays [9, 47]. For species with weak sexual differentiation in fin-ray length and body shape (e.g., *O. pectoralis*), we also assessed visibility of eggs in the ovaries on X-ray images. As an exception, *A. poptae* was included with two possibly immature females (ZMH 22576), which were nonetheless kept due to its distinctiveness and size, being the largest ricefish species.

X-ray images of ricefish specimens were taken using a Faxitron LX-60 cabinet. Lateral radiographs were taken in a strictly plane position, allowing for congruent layering of both lateral sides. This was checked for based on congruent layering of cranial features. Previously obtained maximum intensity projections (two-dimensional renders of three-dimensional models) from available µ-CT Scans (Bruker Skyscan 1172) of fish in planar view were added to the image stack if they fulfilled the criteria for inclusion, i.e. no damage, bending, and clear visibility of relevant skeletal characteristics.

In total, we used 531 individuals from a total of 25 described ricefish species. One of these species, *O. celebensis*, was treated as two separate OTUs (‘Maros’ and ’Rumbia’), since both populations are genetically highly distinct [26, 30, 31]. For the main analyses, males and females were combined (general dataset: “DS-G”); here, only species and additional OTUs with both sexes and genomic data present were used (508 individuals: 22 species, with genetically distinct populations of *O. celebensis* (‘Maros’ / ’Rumbia’) treated as separate OTUs). DS-G was also separately analyzed for each sex in order to investigate whether potential sexual dimorphism may cause divergent results (“DS-G-MF”, with 254 males and 264 females, each with a mean of 11 and 11.7 individuals per species, respectively). In particular, we tested whether intraspecific sexual dimorphism was greater than interspecific variation. Since sexual dimorphism is known to be coupled with ecological differences in diet in some fish species (e.g. in some Telmatherinidae [48]), intraspecific sexual dimorphism exceeding interspecific variation could point towards such a pattern. Further, this comparison was done because sexual dimorphism in ricefishes is known to be disproportionate among species: In pelvic brooding species, females possess derived traits, which are absent in males [49]. These include an abdominal cavity, which could have a significant impact on the mean body shape of a species. The general dataset lacked three species (*A. poptae*, *O. marmoratus* and *O. soerotoi*), which were excluded as only female specimens were available in sufficient numbers. To still take these species into account, we created a female-extended dataset (“DS-FE”). DS-FE comprised 278 female individuals belonging to 25 species, and included juvenile females of the largest known and morphologically highly derived ricefish *Adrianichthys poptae*, as well as adult females of *O. soreotoi* and *O. marmoratus* (Suppl. Tab. S1, S2).

Based on collection data and available literature, information about whether riverine or lacustrine macrohabitats are inhabited was gathered for the Sulawesi-endemic species. Pools, that is small bodies of standing water formed by streams, are also frequently inhabited by ricefishes in Sulawesi [28, 31, 42, 45, 50]. Due to their general dependence on connected flowing waters, we herein consider pools riverine habitats. Species from outside Sulawesi were not assigned to a specific habitat type. At least some better-documented species are widespread, and occurrences in both riverine and lacustrine habitats have been recorded (i.e., *O. latipes*, *O. sinensis*, *O. pectoralis*) [9]. Moreover, certain macrohabitats are difficult to assign to either riverine or lacustrine habitats (e.g., swamps), or may be considered entirely different habitat types (e.g., brackish water estuaries and lagoons). Sulawesi-endemic species are not known from such habitats as of now. Thus, we designated three macrohabitat groups ‘Lacustrine Sulawesi’, ‘Riverine Sulawesi’ and ‘Non-Sulawesi’, and assigned each species to one of them.

### Landmark placement and data collection

**Fig. 2:**
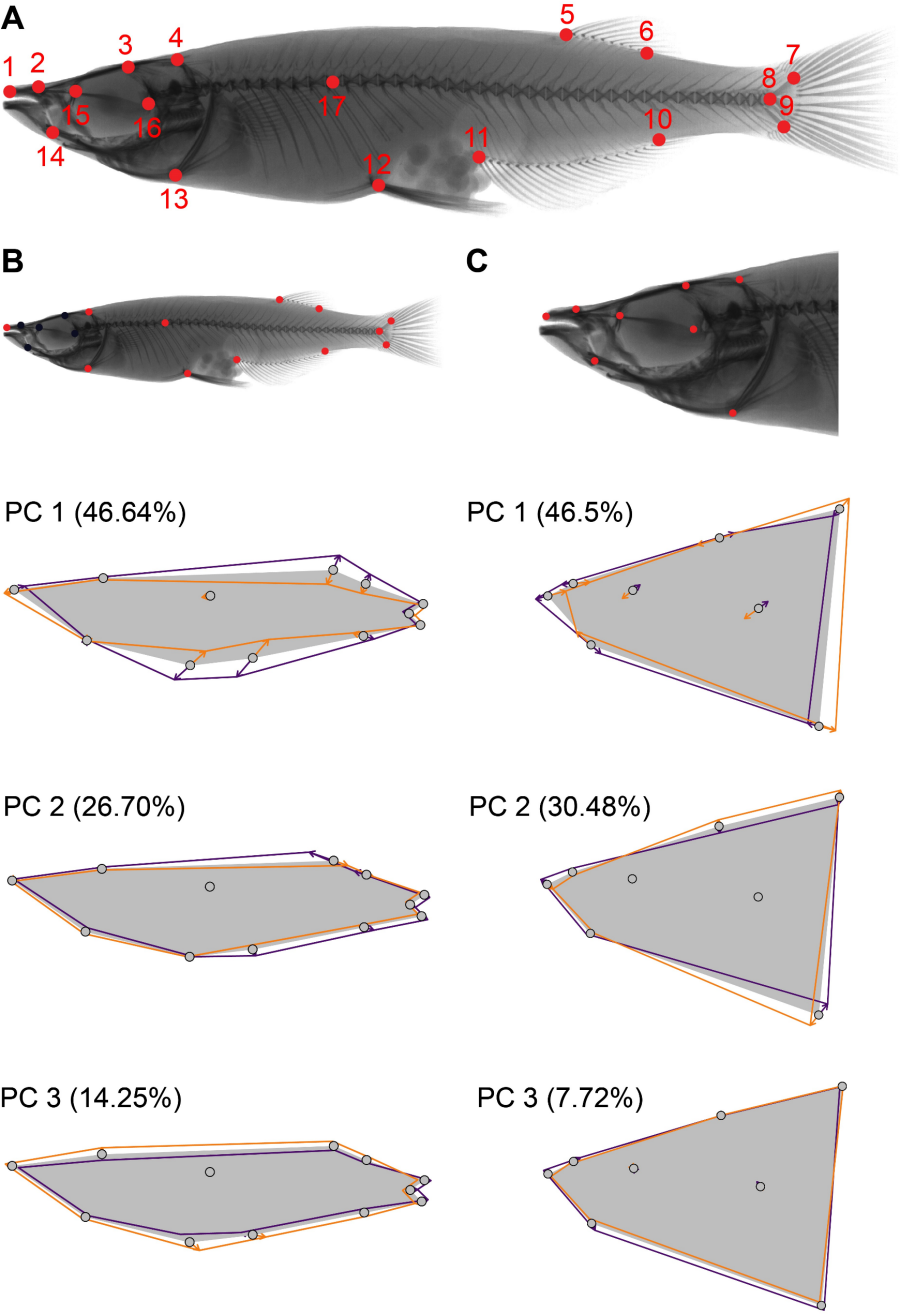
Overview of the landmark placement on skeletal traits (**A**) used to characterize body shape (**B**) and head shape (**C**). Head shape was analyzed separately to avoid masking effects of small but important cranial trajectories by larger body shape differences. Figures below illustrate the shape trajectories identified as explaining the greatest variance in the first and second principal component. Mean shapes of the PC are illustrated as a grey shape, whereas minimum and maximum states along the trajectories are given as orange and purple outlines, respectively. Main axes of variation among species include depth or elongation of body shapes, mouth size and head profiles

Seventeen landmarks for geometric morphometric analyses were placed on homologous skeletal characters that could be reliably identified on the X-ray images (see Suppl. Fig. S1 for definition of each landmark position) for each individual in tpsDig v2.3.2 [51]. All subsequent Geometric Morphometric analyses were performed in R using the packages geomorph v4.0.5 [52] and phytools v1.5 [53]. The main set of landmarks was split into two subsets: Twelve landmarks were selected to represent body shape, while eight landmarks were used to describe head shape, including three (LM 1, 4, 13) shared between both subsets (Fig. 2, Suppl. Fig. S1). The dataset splitting was done to avoid masking effects of highly variable body features on fine cranial features. Subsequently, for each dataset the landmark coordinates were aligned using a generalized Procrustes superimposition analysis (GPA).

Because the degree of sexual dimorphism varies among ricefish species [9, 47, 49], we calculated mean shapes for both sexes of each species, which also corrected effects of disproportionate sample sizes among species and sexes. Subsequently, another Procrustes alignment was performed on the dataset of species-sex group means. For each of the 22 species and additional OTUs with both sexes available in the dataset, we calculated the general mean shape from male and female averages, and performed all analyses and interpretation primarily on this dataset.

### Analysis of geometric morphometric shape data

**Fig. 3:**
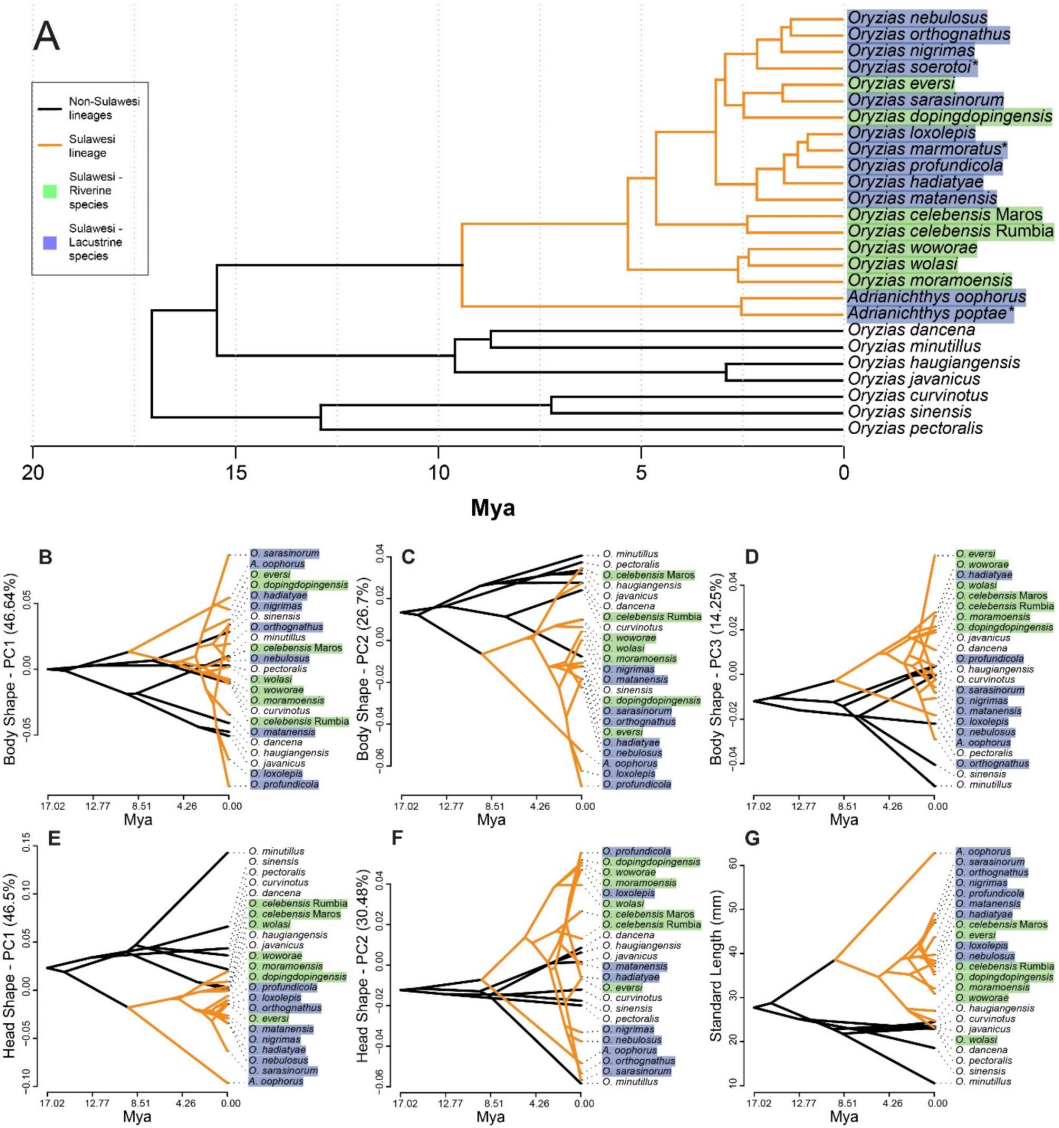
(**A**) Time-calibrated phylogenetic tree for all species included with morphological and genomic data, as retrieved from the analysis of concatenated BUSCO gene set for Actinopterygii (3640 genes) allowing for 30% missing data. Calibration was performed using previously published age estimates based on substitution rates of the mitochondrial *cytb* gene. Phenotypic states, i.e. trait values of extant taxa and state reconstructions of ancestral nodes are presented as phenograms for relevant principal components (explaining > 10% of total variation) of body and head shape, as well as standard length, in the panel below: (**B**) PC 1 of body shape (includes body depth); (**C**) PC 2 of body shape (includes position of dorsal fin); (**D**) PC 3 of body shape (includes position of pelvic and anal fin insertion); (**E**) PC 1 of head shape (includes mouth size); (**F**) PC 2 of head shape (includes head elongation); and (**G**) standard length. Body shape is variable in all groups, but spans a greater range in Sulawesi species. Head shape is slightly less diverse but more derived, whereas standard length is generally larger in Sulawesi species, especially lacustrine ones

To identify shape traits differing the most among species, we performed Principal Component Analyses based on covariance matrices. We examined all principal components (PCs) explaining more than 5% of the total observed variation, but focused on those explaining more than 10%, which we considered informative enough for further visualization. Shape changes along principal components were visualized using Backtransform Morphospaces [54], which add a grid pattern of shape outlines representative of the shape states at the plotting coordinates to the morphospace plot (Fig. 3), as well as using spline plates (Fig. 2).

### Disparity

Shape disparity was estimated using the Procrustes ANOVA approach in geomorph (*morphol.disparity*), which utilizes the coordinates of the General Procrustes Alignment to calculate overall and group-specific Procrustes variances and performs pairwise comparisons between groups, in our case between the macrohabitat type (‘Riverine’, ‘Lacustrine’ and ‘Non-Sulawesi’) to identify how disparate they are from each other. To assess the contribution of each of these groups to the total disparity observed for the dataset, we calculated Foote’s partial disparities, for which the sum of group partial disparities equals the total disparity of the dataset [52, 55].

### Phylogenetic analyses

Using published whole-genome short read data from [26, 30], and reference genome assemblies of *O. javanicus*, *O. latipes* and *O. sinensis* (Suppl. Tab. S1), we created a phylogeny based on the BUSCO gene set for Actinopterygii [56] (actinopterygii_odb10, busco.ezlab.org). A previous study pointed out that the taxon identified as *O. carnaticus* in [26, 30] is most likely *O. dancena* [32]. We agree with this conclusion and follow it here. Consequently, we used available reads of specimens misidentified as *O. carnaticus* for our assembly of *O. dancena*, as specimens used in our geometric morphometric analyses and the individual used for WGS both originate from India, whereas other data available for this widespread species [9, 57] is based on strains of unknown or more distant geographic origin.

To improve the accuracy of the phylogeny, also species that could not be included in the geometric morphometric analyses were retained. The reference genome of *Xiphophorus maculatus* (GCA_000241075.1) was chosen as a single outgroup to root the phylogeny. Whole-genome reads were assembled using SPAdes v3.15.0 [58] with default settings. A set of single copy orthologs was identified with Buscophy (Martin et al., in prep, https://gitlab.leibniz-lib.de/smartin/buscophy), a custom in-house workflow tool utilizing the workflow management system Snakemake [59]. In a first step, genes from the Actinopterygii BUSCO set (actinopterygii_odb10) were extracted from each assembly and saved in alignments with BUSCO v5.5.0 [60], and sequences subsequently were assigned to orthologous groups. From the 3640 genes included in the BUSCO set for Actinopterygii, between 2172 and 3543 genes were recovered as complete among the assemblies and between 60 and 929 genes as missing (Suppl. Fig. S2). Translated amino acid sequences were then aligned with MAFFT v7.520 [61, 62], and a corresponding nucleotide alignment was generated with PAL2NAL v14 [63]. After a filtering step with trimAl v1.4.rev15 [64], alignments of each gene were concatenated into a single supermatrix with AMAS [65] with third codon position masked. Maximum likelihood phylogenies were reconstructed using substitution model GTR + I + G with IQ-TREE v2.1.2 [66] with 1000 bootstrap pseudoreplicates allowing for missing data (proportion of missing taxa in each individual BUSCO gene sequence alignment) between 0 − 50% (in 5% steps). Retrieved topologies were compared to those of published molecular phylogenetic studies [26, 27, 30], which show a high degree of consistency, as well as population genomic studies focusing on selected species groups [31, 39, 67–71]. The goal was to retrieve a dataset with as little missing data as possible while retaining a topology that overall matches the published ones best. Following this, we used the dataset with 30% missing data (2757 genes, 4905300 bp) and the resulting topology for subsequent phylogenetically informed analyses. The retrieved phylogenetic tree was ultrametricized using the *chronos* function of the R package ‘ape’ version 5.8 [72]. For the calibration of the chronogram, we used upper and lower limits of divergence time estimates published by [32] based on substitution rates of the mitochondrial *cytb* gene, which in turn were retrieved from divergence times of major lineages of Japanese *Oryzias* [12, 29]. We only used age estimates for the two oldest nodes representing the splits of the three main monophyletic groups of Adrianichthyidae sensu [29]. Namely, these include the split of the *O. latipes* group, estimated to have diverged between 13.1 and 20.8 Mya, and the split of *O. javanicus* and *O. celebensis* sister groups, estimated to have occurred between 11.4 and 18.2 Mya [32]. Finally, the phylogeny was pruned of the outgroup *X. maculatus* and ricefish species without available shape data, leaving 24 described species (with two OTUs for *O. celebensis*) (see Suppl. Table 1).

### Phylogenetic signal

Apart from visual examination of the PCA results, we also investigated the impact of phylogenetic signal on our data, and compared results for Sulawesi and non-Sulawesi groups. We calculated Blomberg’s K [73] to estimate the strength of phylogenetic signal, which compares the association between phenotypic similarity and phylogenetic distances relative to expectations under a Brownian motion model of evolution. Here, greater values imply greater shape divergence independent of phylogeny. K, the ratio between observed and expected phenotypic variance, is expected to be 1 under Brownian motion, while values < 1 or > 1 are associated with higher or lower shape diversity, respectively. The null hypothesis K = 0 suggests no phylogenetic signal and therefore no correlation of phenotypic similarity and phylogenetic relationship. The function was performed with 1000 random permutations on the Procrustes coordinates of the mean shapes for significance testing.

### Phenograms, phylomorphospaces and ancestral state reconstructions

In order to visualize the shape diversity of ricefishes in relation to their phylogeny, comparing the Sulawesi lineage to all non-Sulawesi species in particular, we created phenograms, which plot the chronogram in a way that each species tip in the phylogeny corresponds to a specific trait value along the y axis. As trait values, we used the score ranges of all Principal Components in both body and head shape explaining more than 10% of the total variation, as well as species means of standard length. Phenograms were created using the *phenogram* function of the R package ‘phytools’.

We further plotted phylomorphospaces for species means of body and head shape [74], using the *phylomorphospace* function of R package ‘phytools’. This visualizing method draws regular shape coordinates in the principal component morphospace, which are connected by branches and nodes following the topology of a provided phylogeny. Thereby, these plots are informative about the approximate evolutionary history of morphospace colonization. Ancestral nodes of the phylogenetic topology are positioned relative to the branch lengths connecting them, thereby providing ancestral state estimates. Further, information about cases of repeated shape evolution is provided, i.e., if distant tips of the phylogenetic tree cluster in close proximity to each other.

### Rates of shape evolution

To assess how rapidly Sulawesi ricefishes, and lacustrine species in particular, have diversified in shape, we compared net rates of phenotypic shape evolution. To do so, we used the macrohabitat assignment from our species data frame, namely lacustrine Sulawesi, riverine Sulawesi and non-Sulawesi ricefish species (see Suppl. Table 1). We then calculated evolutionary rates of body and head shape evolution with the function *compare.evol.rates* of the R package ’geomorph’ and compared retrieved rates. For significance testing, 1000 random permutations were calculated.

### Body size

Standard lengths vary noticeably among ricefish species. Shape is commonly correlated with size, and species differences may also be affected by phylogeny, termed evolutionary allometry [75]. In contrast, deviations in allometry can indicate alternative underlying drivers of phenotype evolution such as ecological adaptations [76]. Since only species means were included, which in turn are based on adult specimens exclusively, intraspecific allometry was omitted. Standard length, taken from the tip of the snout to the end of the hypural plate, was used to represent body size irrespective of caudal fin length. To test whether deviations are present, or differences in shape are merely reflecting size, we used direct measurements of standard length, and plotted regression scores of body shape (Procrustes coordinates) on mean standard lengths for each species against mean standard length alone. Subsequently, allometric deviations within and among phylogenetic lineages and habitat types were compared visually by creating a phylomorphospace.

## Results

### PCA

**Table 1:** Relative proportion of the total variation explained by principal components recovered in PCAs of body and head shape, respectively

	PC 1	PC 2	PC 3	PC 4
**Body Shape (%)**	46.64	26.7	14.25	5.34
**Head Shape (%)**	46.5	30.48	7.72	5.26

**Fig. 4:**
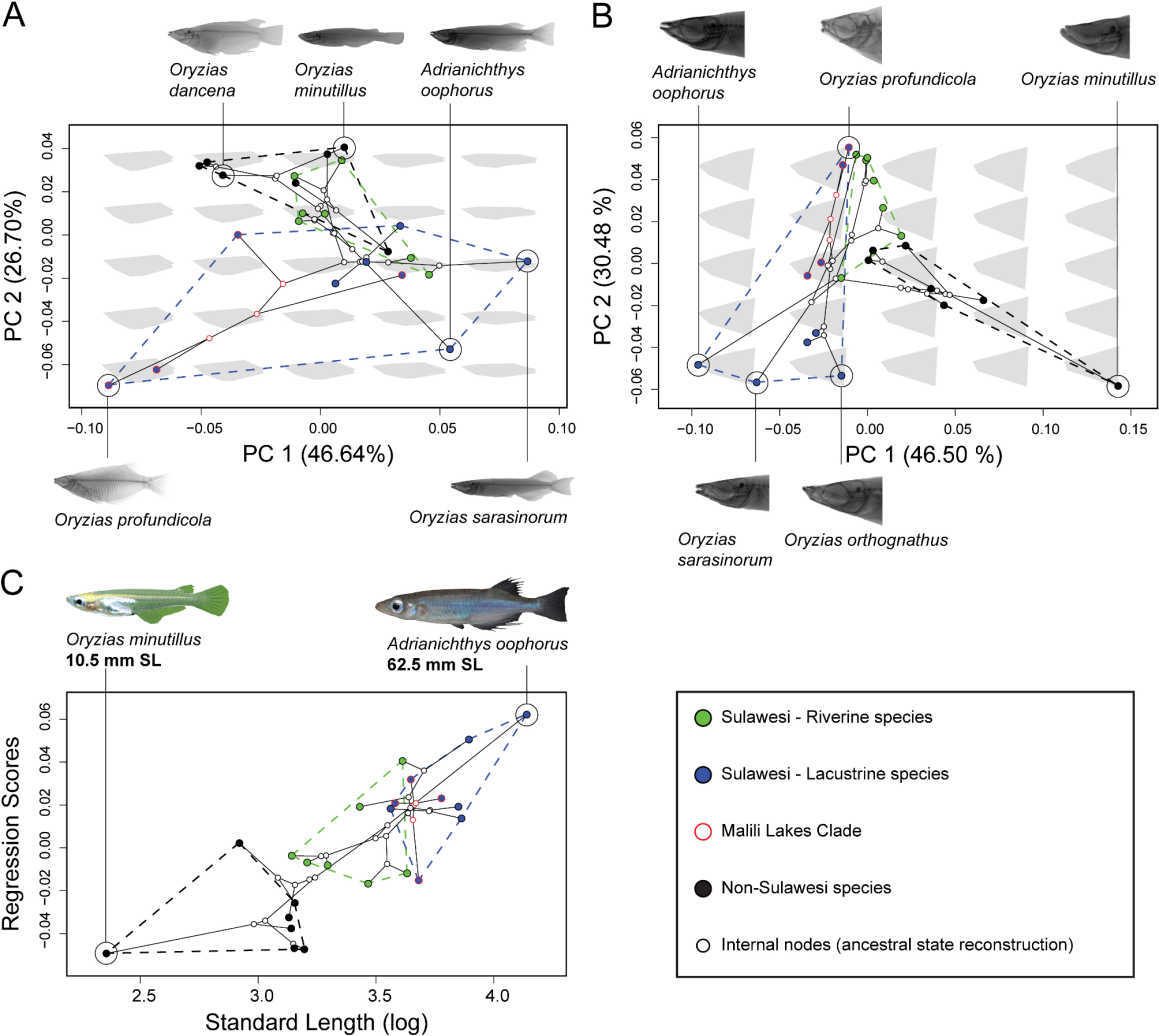
Phylomorphospaces of (**A**) PC 1 and PC 2 of body shape; (**B**) PC 1 and PC 2 of head shape (trajectories of each PC are as described for Fig. 2), and (**C**) regression of body shape (Procrustes coordinates) on standard length versus standard length alone. The phylogeny recovered using concatenated BUSCO genes is projected into the morphospace (see Sidlauskas 2008). Taxonomic entities are color coded by macrohabitat group; blue = lacustrine; red = riverine; black = non-Sulawesi. Internal nodes of the phylogeny are colored white and provide estimates for ancestral states. Lacustrine and riverine habitat groups show clear disparity, despite close phylogenetic relationships. Red outlines indicate species tips and internal nodes of the clade restricted to the Malili lakes system, which was found be particularly diverse in body shapes. Riverine species are generally more similar to non-Sulawesi species, whereas lacustrine species readily colonized previously unoccupied morphospace regions. Backtransform morphospaces of the respective PC morphospaces are plotted as grey shapes into the background. These are based on convex hulls of landmark constellations, illustrate shape changes along the PC axes and represent body shape states at the plotting coordinates. Refer to Suppl. Fig. S3-5 for species labels of all data points

A PCA on shape data revealed the most prominent shape differences among species (Table 1). For PC1, we observed mostly changes in dorsal, anterior anal and pelvic fin insertions (LM 3, 4, 9 and 10), points that correspond to body depth (Fig. 2). The extremes of PC 1 morphologies range from slender *Adrianichthys* from Lake Poso, to deep-bodied *Oryzias loxolepis* and *O. profundicola*, both from Lake Towuti (Figs. 3B-D and 4A). PC2 mainly describes differences in the insertion of the dorsal fin (landmarks 3 and 4, Fig. 2). Among lacustrine species, there is a tendency towards more posterior insertions (Figs. 2 and 4A), contrasted by riverine ricefishes with more anterior insertions. PC3 comprises changes associated with a deeper pelvic fin insertion, corresponding to a deeper ventral body, but without simultaneous elongation as in PC2 (Fig. 2B).

Similar to body shape, PC1 of head shape comprises changes in head depth, including snout elongation and depth expansion of the posterior head (Fig. 2), but also general mouth size. Among all species included, *Adrianichthys* possess the most elongated heads. There is a general tendency for lacustrine species to have more elongated heads and larger mouths (Fig. 3b). In contrast, riverine and non-Sulawesi species possess much less elongated and rather deep heads. PC2 includes changes associated with a more elongated snout, larger lower jaws, as well as a more anterior tip of the cleithrum (Fig. 2, LM 13). Here, non-Sulawesi species are more confined to the negative extremes, whereas no clear separation between lacustrine and riverine habitat groups is apparent. Still, the former groups covers a much larger trait range due to the phenotypes with elongated snouts, which are absent from the riverine group (Fig. 4b).

Among adrianichthyids, interspecific disparity is apparent in body and head shapes. In all morphospaces composed of relevant PC axes, lacustrine Sulawesi ricefishes occupy more space than riverine and non-Sulawesi species, as well as those two groups combined (Fig. 4, Suppl. Fig. S3-4). The extremes of the morphospace occupation include mostly lacustrine Sulawesi ricefish species, expanding deep into areas unoccupied by the other two groups. Particularly large variation is apparent for PC 1 and 2 of body shape and PC 2 of head shape within the Malili Lakes species group. The Malili lakes are a system of larger (Lakes Matano, Mahalona and Towuti) and smaller (Lakes Lantoa and Masapi) lakes connected by rivers (see e.g. [19] for more details). Of this species group, *O. matanensis* from Lake Matano, *O. loxolepis* and *O. profundicola* from Lake Towuti (also *O. marmoratus* from Lake Towuti in the female-specific analysis), and *O. hadiatyae* from Lake Masapi where included in the analysis (Fig. 1). Riverine ricefishes cluster closer to the non-Sulawesi species included, particularly in body shape, and to a lesser extent also in head shape. Notably, the lacustrine group contains several closely related (evolutionarily young) but phenotypically disparate lineages (e.g., *O. hadiatyae* and *O. marmoratus*), while phylogenetic distances of riverine species are large between clades despite being morphologically similar (e.g., *O. celebensis* and the clade containing *O. moramoensis*, *O. wolasi* and *O. woworae*).

In the PCA results on the female extended dataset (DS-FE), which are highly similar to those of the general dataset (DS-G), the extend of head shape morphospace occupied by lacustrine species increased even more drastically due to the inclusion of *A. poptae*, the largest and most elongated ricefish species (Suppl. Fig. S6-8).

### Sexual dimorphism

PCA of species-sex means shows that sexual dimorphism in body shape is, as expected, generally lower than interspecific variation (males and females clustered in close proximity in PCA plots; Suppl. Fig. S10). Trajectories of shape variation recovered in sex-specific PCAs (Suppl. Fig. S9) largely match those of the main analysis based on species means, including the relative contribution of the first three PCs to total variation. Generally, males tend to be slightly more deep-bodied than females, but also show a more anterior dorsal fin insertion. As expected, sexual dimorphism in body shape is pronounced in pelvic-brooding species, where females possess specialized morphological adaptations for long-term egg carrying, such as a ventral concavity, which influence shape. Still, both the distances and trajectory direction between sexes of pelvic brooding species in the PCA morphospaces do not differ substantially from those of many transfer brooding species, which demonstrates that this influence is limited in our dataset. Larger distances in head shape between females and males are observed in *O. celebensis* ‘Rumbia’, but not in *O. celebensis* ‘Maros’, along PC 2 (Suppl. Fig. S11). Most likely, these differences are the result of the low male sample size in the former OTU (2 males, 5 females). Apart from this, major sexual dimorphism in head shape is not evident, although males generally possess bulkier heads, as seen in greater intersexual differences along PC2. Except for *O. nebulosus* and *O. profundicola*, where males grow slightly larger than females (38.3 vs. 32.2 mm SL and 46.4 vs. 41 mm SL, respectively), and in *O. hadiatyae*, where males remain slightly smaller than females (35.7 vs. 41 mm SL), sexual dimorphism in standard length was not found (difference between sexes < 10%).

### Group disparities, phylomorphospace and ancestral state reconstructions

For body shape, Procrustes variances are highest in the lacustrine species group and lowest in the non-Sulawesi group (Table 2). For head shape, Procrustes variances are highest in the riverine group and lowest in the non-Sulawesi group. Significant differences in morphological disparity in body and head shapes were not recovered for any pairwise comparison of macrohabitat groups (Table 2). Foote’s partial disparity is clearly highest for the lacustrine group in body shape. It is highest for the riverine group in head shape, although being comparable to that of the lacustrine group. For both body and head shape, lowest partial disparity was recovered for the non-Sulawesi group (Table 2).

**Table 2:** Procrustes variances as a measure of morphological disparity in body and head shape within riverine and lacustrine sulawesi groups and non-sulawesi group. Below, pairwise distances between partial disparities of groups. Upper triangle shows pairwise absolute differences among group Procrustes variances, lower triangle p values. An asterisk marks significant disparities (*p* < 0.05)

		Riverine	Lacustrine	Non-Sulawesi
**Body shape**	Procrustes variance (Foote 1993)	7.437 × 10^− 4^ (19.93%)	1.801 × 10^− 3^ (48.25%)	1.188 × 10^− 3^ (31.82%)
	**Riverine**	**-**	1.057 × 10^− 3^	0.444 × 10^− 3^
	**Lacustrine**	*0.100*	**-**	0.613 × 10^− 3^
	**Non-Sulawesi**	*0.423*	*0.371*	**-**
**Head shape**	Procrustes variance (Foote 1993)	2.118 × 10^− 3^ (46.23%)	1.904 × 10^− 3^ (41.55%)	5.596 × 10^− 4^ (12.22%)
	**Riverine**	**-**	0.214 × 10^− 3^	1.558 × 10^− 3^
	**Lacustrine**	*0.890*	**-**	1.344 × 10^− 3^
	**Non-Sulawesi**	*0.077*	*0. 236*	**-**

In the phylomorphospace of body shape, the range of shape states of riverine Sulawesi species is more similar to the non-Sulawesi group than the more extensive range of lacustrine Sulawesi species. In head shape, riverine species are intermediate to non-Sulawesi and lacustrine ricefish species (Fig. 4b). In PC2, which includes head elongation, the lacustrine group extends towards both extremes. Riverine ricefishes have more stout head shapes, with limited overlap with non-Sulawesi species (Figs. 2 and 4b). Internal nodes of the phylogeny were reconstructed as having less extreme trait states, congregating around the non-Sulawesi species with the exception of PC1 of head shape (Fig. 4).

The phenograms agree with results from phylomorphospaces and show clear differences in trait variance among different lineages (Fig. 3). While body shape varies also in non-Sulawesi lineages, differences are more pronounced in Sulawesi species and evolved in much shorter time. In contrast, head shape, and also standard length, differ greatly between Sulawesi species and non-Sulawesi counterparts. Ancestral states of the common ancestors (internal nodes) were reconstructed to be intermediate in trait values for body and head shape. In addition, standard lengths were rather small, as opposed to Sulawesi ricefishes (Figs. 3G and 4C, Suppl. Fig S5).

### Phylogenetic signal

As a measure for phylogenetic signal in our dataset, K values < 1 were recovered for body shape (K = 0.318, *p* = 0.017) and head shape (K = 0.448, *p* = 0.001) in the datasets of species means, which corresponds to less phylogenetic influence in shape data than expected under a Brownian motion model of evolution. Comparing signal strength between sexes, the recovered signals are similar among males and females for body shape (K = 0.305, *p* = 0.019, and K = 0.329, *p* = 0.006 respectively), and slightly higher for head shape in females (K = 0.464, *p* = 0.001, compared to K = 0.416, p = 0.001 for males).

Additional group-specific tests for phylogenetic signal in the dataset revealed low values for Sulawesi species in body and head shape datasets. Both for body and head shape, lower phylogenetic signal was recovered for the Sulawesi group (K = 0.47, *p* = 0.088, and K = 0.61, *p* = 0.014 respectively) than non-Sulawesi group (K = 0.869, *p* = 0.07, and K = 0.725, *p* = 0.348 respectively).

### Rates of shape evolution

Estimates for the rate of body shape evolution revealed significant differences to a Brownian motion model of evolution. Rates are highest in lacustrine species, followed by riverine and non-Sulawesi species. In particular, the estimated rate for lacustrine species is greater by a factor of more than three compared to riverine species (Table 3). Similar results were recovered for head shape. For the non-Sulawesi group included in this study, we recovered rates of body and head shape evolution closer to, but still lower than those of riverine Sulawesi ricefishes.

**Table 3:** Net rates of body and head shape evolution (Sigma D) for the riverine and lacustrine Sulawesi groups and non-sulawesi group under a brownian model of evolution after phylogenetic transformation. The observed rate ratio gives the ratio of maximum to minimum net evolutionary rate

	Species Means				
	*Observed rate ratio*	*p*	*Sigma D Lacustrine*	*Sigma D Riverine*	*Sigma D Non-Sulawesi*
**Evolutionary rate of body shape**	11.115	0.0001	6.481 × 10^− 5^	1.725 × 10^− 5^	5.831 × 10^− 6^
**Evolutionary rate of head shape**	3.994	0.0003	7.729 × 10^− 5^	2.966 × 10^− 5^	1.935 × 10^− 5^

### Body size

Compared to non-Sulawesi ricefish species, Sulawesi ricefishes cover a wide range of standard lengths, and are generally larger (Figs. 3G and 4C, Suppl. Fig. S5). Plotting of residuals of the shape-size regression against standard length revealed pronounced allometric separation of lacustrine and riverine Sulawesi ricefishes. Species with a greater standard length are generally lacustrine, while smaller ones are mostly riverine or non-Sulawesi species. The smallest (*O. minutillus*) and largest known ricefish species (*A. poptae*) are a non-Sulawesi and a lacustrine species, respectively.

## Discussion

### Greater phenotypic diversity in Sulawesi ricefishes compared to continental species

Crossing biogeographical barriers, such as Wallace’s line, can provide organisms with access to novel ecological opportunities. Ecological opportunity itself is known to promote occupation of new ecological niches and rapid phenotypic evolution [2, 4, 77]. In fishes, shapes of body and head serve as proxies for habitat and trophic specialization, swimming performance and maneuverability, or predator avoidance [78–80]. As phenotypes are greatly affected by ecology and function [81], shape and size data are useful proxies to investigate the degree of diversification and the potential influence of ecological opportunities experienced by ricefishes in Sulawesi.

Based on the published literature, it appears that Sulawesi ricefishes show a wider range of shape and size than non-Sulawesi ricefishes [9, 13]. In agreement with this, we find Sulawesi species to be phenotypically more diverse than non-Sulawesi species. Although there is overlap between the Sulawesi and non-Sulawesi groups, the areas in the morphospace occupied exclusively by the Sulawesi ricefish group are larger than those of the non-Sulawesi group, both in terms of body and head shape (Fig. 4). This increase in shape diversity translates to more derived novel shape phenotypes, which effectively colonized previously unoccupied morphospace areas. Such expansion is observable especially along trajectories related to body depth or elongation, mouth size and head profiles (Figs. 2 and 4). The observed general patterns in phenotypic diversity and rates of shape evolution in Sulawesi ricefishes (Fig. 3; Table 3) also suggest stronger evolutionary drivers like those related to adaptive mismatch-based niche discordance [3]. Although it was shown that adaptive diversification does not necessarily require coupling of taxonomic and phenotypic diversification, its simultaneous occurrence is a strong indicator that ecological opportunity played a major role during diversifying evolution [77, 82, 83].

In addition to general diversification patterns, there appears to be substantial disparity in head shapes between Sulawesi and non-Sulawesi ricefishes, where the former possess relatively larger mouths (Figs. 2 and 4). This might reflect general ecological release on the island from major oriental freshwater fish lineages such as cypriniforms [24, 25], which allowed the occupation of trophic niches similar to those of more competitive lineages in Sulawesi. In addition, Sulawesi ricefish species tend to be larger than their non-Sulawesi counterparts (Figs. 3G and 4C), which might equally be explained by ecological release due to a reduced number of predatory or competing lineages. This represents a stark contrast to a range of miniature species known from mainland Asia, such as *O. minutillus* and *O. mekongensis* [9, 57]. These miniature species, defined as not reaching lengths greater than 25 mm SL [9, 84], are not monophyletic and evolved repeatedly in different lineages despite considerable morphological similarity [9, 30, 57]. Although insufficiently sampled in our study, they appear to be morphologically very distinct from other ricefishes. In our dataset *O. minutillus* greatly extended the morphospace occupation of non-Sulawesi ricefishes in various plots (Fig. 4, Suppl. Fig. S3-5). True miniature ricefish species are not known from Sulawesi at present, and no Sulawesi species clustered in close relative proximity in our morphospace analyses. Miniaturization in freshwater fishes is known especially from nutrient-poor and spatially reduced microhabitats with still or slow-flowing water [84–87], but can also represent a coping strategy for competition or predation pressures [88–91]. However, freshwater habitat types in Sulawesi occupied by ricefish also include large lakes and streams, which in oriental regions are usually dominated by other fish lineages absent from Sulawesi [9, 24, 25]. It is therefore conceivable that release from competition or predation would also release them from selection pressures promoting a reduction of body size. Altogether, size diversification largely followed opposite trajectories in Sulawesi, where conditions enabled large body sizes, and mainland Asia, were miniaturized phenotypes evolved multiple times independently.

Finally, conspicuous coloration also appears to be more common in Sulawesi ricefishes compared to non-Sulawesi ricefishes. While coloration itself is difficult to quantify, bright body coloration of certain species, as seen for example in *O. woworae* and *O. profundicola*, is only known from Sulawesi ricefish species, which could also be explained by ecological release from competition and predation.

### Phenotype diversification in lacustrine Sulawesi ricefishes

In Sulawesi, the phenotypic range of lacustrine ricefish species expanded far beyond the extremes of riverine species, corresponding to our hypothetical scenario of generally augmented shape diversity and providing a line of evidence for ecological opportunity as an evolutionary driver of diversification. Body and head shape disparities are greatly increased along multiple trajectories (i.e., both more slender and deeper bodies) in lacustrine species, indicative of multiple different drivers for diversification. Several shape shifts, especially in head shape, suggest distinct selection pressures related to trophic ecology. Elevated evolutionary rates in the lacustrine species group compared to riverine group indicates stronger ecological selection, favoring less common phenotypic states and leading to shifts in morphospace occupation [92].

Although there are currently more lacustrine than riverine ricefish species known from Sulawesi, phylogenetic effects, while present, do not explain the observed disparities sufficiently and phylogenetic signals are generally lower than expected under a Brownian motion model of evolution. Moreover, there are major differences in rates of shape evolution between the two habitat groups. This is in line with expectations under adaptive diversification, because stronger phylogenetic signal should be retrieved if species richness evolved mainly due to allopatric speciation under similar selection pressures [93]. The observed pattern supports the assumption that shape diversification was promoted by macrohabitat with links to differential trophic and microhabitat niches. In comparison, riverine species were rather similar to each other, particularly in head shape (Fig. 4b), implying a limited role of trophic niche occupation. In agreement with views that riverine habitats hold limited potential for ecological diversification [35, 94], body size is also more confined despite deeper phylogenetic divergences. The largest species in our dataset are all exclusively lacustrine, suggesting that ecological conditions in lake environments may promote increases in body size, via potential candidate factors linked to ecological opportunity, such as trophic specialization or food availability, or other factors like predation. While we do not rule out selection pressures that also promoted adaptive trait shifts in riverine ricefishes, they are most likely masked by much more substantial differences in lacustrine species in our analyses.

### Niche–shape association and drivers of phenotypic evolution

Phenotypic diversification upon colonization of lake environments offering ecological opportunities has been recorded in various freshwater fish lineages [79, 94–96]. Lake size was found to influence species richness in African cichlids, in part by providing more exploitable resources and thus increased ecological opportunity [35, 97]. Under such conditions, trophic specialization to narrowed-down niches is a common trajectory of diversification. In Sulawesi ricefishes, there is clear evidence that colonization of lakes was a major driver of phenotypic diversity. Sympatric ricefish species, that is species pairs or small species flocks, are also known to exist in several lakes. Adaptive diversification is well-known from lake flocks, and commonly manifests itself in shape disparities between species [79, 98–101].

A particularly prominent vector of morphospace expansion in lacustrine Sulawesi ricefishes is body depth. Elongation and deepening of body shape have profound effects on the locomotion of fish, and are commonly linked to habitats [102]. In our dataset, ricefish species with pelagic ecologies (*O. sarasinorum* and *A. oophorus* [9, 25]), exhibit the most elongated body shapes. Commonly seen in fish species cruising in open waters, elongation, i.e. a decrease of body depth, decreases drag by reducing body area, while a tapered head and narrow caudal peduncle further help to maximize thrust and minimize energy loss. Likewise, these traits can also be observed in pelagic ricefish species, including an example of shape homoplasy between distantly related taxa with similar ecologies (*Adrianichthys* and *O. sarasinorum*). Increases in body depth on the other hand improve maneuverability by optimizing the energy efficiency of movements involving a rotation of the vertical body axis, e.g. swimming turns. Hence, deep body shapes are commonly seen in mobile fish from geometrically complex environments. Of the sympatric *Oryzias* species in Lake Poso, *O. nebulosus*, which occurs in three dimensionally complex scree habitats, shows the deepest body shape. While deep, diamond-shaped bodies are characteristic of many ricefish species, a rather extreme version of this phenotype can be seen in *O. profundicola*, as well as the closely related and sympatric *O. loxolepis* [39] from Lake Towuti. In particular, deep-bodied ricefishes have been reported to frequent deeper waters of the lake ( [103]; Herder, pers. obs.; Mokodongan et al., pers. obs.), were also predatory fish such as large sailfin silversides (*Paratherina* spp.) and gobies (*Glossogobius* spp.) are present [19, 25]. Thus, it is possible that deeper bodies evolved in Lake Towuti ricefish as a response to colonization of water layers with higher density of piscivore fish. Here, deeper bodies may help reduce predation by exceeding the width of a predator’ mouth gape, and improving maneuverability to help escape predators.

In addition to body shape-related phenotype diversity, head shape is another vector of morphospace expansion. Also here, a prominent vector comprises variation in head length. However, in many cases changes in head length may also have evolved as adaptation to locomotion phenotype. Indeed, extremely elongated head shapes are present in the same species that also show the greatest body elongation. Moreover, it is much more difficult to discuss a possible link to trophic specialization given a general lack of data on in-situ diet. Nonetheless, the most distinct (i.e. extreme) head shapes were found for species belonging to the two sympatric species flocks inhabiting Lake Poso, namely the Lake Poso *Oryzias* lineage (*O. nebulosus*, *O. nigrimas*, *O. orthognathus*) and the endemic genus *Adrianichthys* (Fig. 3F, Suppl. Fig. S4, S8). Lake Poso is the second-largest lake in Sulawesi, and its endemic ricefish species are phenotypically well-differentiated from each other and commonly show derived head shape traits like elongated snouts in *Adrianichthys* species or enlarged lower jaws in *O. orthognathus*. Since head shape is directly linked to trophic adaptation [104, 105], this strongly suggests trophic niche partitioning or specialization as an evolutionary driver of diversification, with support coming from ecomorphological and stable isotope data [106]. Interestingly, in sympatric ricefish species in Lake Towuti (*O. marmoratus*, *O. loxolepis* and *O. profundicola*), only weak differentiation in head shape is apparent, contrasted by more pronounced body shape disparity (Fig. 4A, B; Suppl. Fig. S3-4, S7-8), indicating that in this case trophic differentiation was possibly a subordinate driver of diversification. Apart from general head elongation, there is also a tendency for larger mouth opening in lake species, which indicates increases in prey size limit.

The existence of sympatric species in Lake Towuti suggests that the presence of other diversifying fish lineages, such as sailfin silversides (Telmatherinidae) did not completely prevent the diversification of ricefishes into multiple phenotypically differentiated ecomorphs. Studies on the evolutionary age of sailfin silversides suggest that they colonized Sulawesi and diversified several million years prior to ricefish [15, 107]. However, it remains entirely possible that predation by and competition with these lineages constrained further diversification of ricefishes in Lake Towuti. This is supported by a lack of open-water dwelling species, like those known from Lake Poso (*Oryzias orthognathus*, *Adrianichthys* spp.) and Lake Lindu (*Oryzias sarasinorum*), both lakes from which other radiations of fish lineages have not been described.

Apart from expansion into novel regions of shape space, large unoccupied regions in the morphospace were also present within them, suggesting evolutionary bias towards certain shape states. Particular examples include a lack of deep-bodied species with relatively posterior dorsal fins, possibly reflecting constraints in swimming performance. There are also no large species, herein defined as species known to grow larger than 50 mm SL, with deep bodies. In addition, we found no species with stout head shape in combination with either short-snouted superior or long-snouted terminal mouth (Fig. 4). Instead, stout head shape is exclusively associated with intermediate states (short snout, terminal or slightly superior mouth). Further, there are indications of functional constraints through trophic morphology: no benthic feeding modes are known from extant species, and only the two presumably extinct *Adrianichthys kruyti* and *A. roseni* have subterminal mouths, which morphologically could indicate a possibility for such a feeding mode [9, 33, 34]. In fact, the default mode in ricefishes seems to be planktivory, and utilization of prey resources might become more functionally difficult to achieve with increasing prey size.

### Sexual dimorphism

Despite representing an important influence on phenotypic evolution, sexual dimorphism is frequently neglected in taxon-specific diversification studies. While strong divergent selection is seen as the dominant driver of phenotypic differentiation, sexual selection and resulting sexual dimorphisms may add additional complexity to phenotypic diversity of fish radiations [48, 99, 108]. Sexual dimorphism may occur in ecologically relevant traits, with ecological differentiation and character displacement [48, 49, 109, 110]. We find that interspecific variation in body shape of Sulawesi ricefishes is more pronounced than sexual dimorphism, which is generally confined to males being slightly more deeper-bodied than females. We did not recover clear sexual dimorphism in head shape in any species, providing no evidence for sexual dimorphism in resource use. Nonetheless, sexual dimorphism in body shape is pronounced in pelvic brooding species due to the presence of a ventral concavity in the pelvic region, in which female fish carry fertilized egg clusters until hatching [13, 27, 33, 49, 111]. Since the three most slender pelvic brooding species, *A. oophorus*, *A. poptae* and *O. sarasinorum*, are pelagic lake fish [9, 13, 25, 28, 49], pelvic brooding as a novel reproductive ecology appears to have at least facilitated the occupation of open water by circumventing the necessity to enter littoral microhabitats for spawning.

### Colononization of Sulawesi and exposure to ecological opportunity

Since Sulawesi, a composite island formed by fusion of volcanic and old continental fragments [112], was never connected to larger landmasses, it is most likely that ricefishes colonized the island via marine waters. Indeed, phylogenetic evidence suggests an ancestor of Sulawesi ricefishes related to the extant *O. javanicus* group, which includes several species with hyperosmotic tolerance commonly found in brackish waters (e.g. *O. carnaticus*, *O. javanicus*, *O. dancena*, *O. haugiangensis*) [9]. However, in contrast to other freshwater radiations originating from marine ancestors, such as ariid catfishes or pufferfishes [113, 114], there are no truly marine ricefish species, as all are known to occur in pure freshwater [9, 115]. Still, this osmotic tolerance allowed them to cross a marine barrier that remained impassible for most other freshwater fish lineages, including dominant groups of the Oriental region such as cypriniform and siluriform fishes. Apart from ricefishes, only some halfbeak (Zenarchopteridae: *Dermogenys*, *Nomorhamphus*), sailfin silversides (Telmatherindae: *Telmatherina*, *Paratherina*, *Tominanga*) and goby (Gobiidae: *Glossogobius*, Oxudercidae: *Mugilogobius*) lineages have evolved into exclusively freshwater-inhabiting fish lineages comprising multiple species in Sulawesi [25]. At present, there is no evidence that Sulawesi was once inhabited by other such lineages. The age of the Sulawesi ricefish radiation has been the subject of several studies, with widely differing estimates of colonization time points ranging from 4 to 47 Mya [12, 15, 29, 30, 32]. Recent data from substitution rates of mitochondrial genes, on which the calibration of our chronogram is based, suggests a more intermediate age of ~ 14.6 Mya [32]. This is similar to estimates for the colonization of Sulawesi by a clade of sailfin silversides, which were found to have split from a brackish-marine sister species between 8.5 and 28.9 Mya [107].

Given the isolation of Sulawesi, a majority of its freshwater fish species are amphidromous, meaning that they require access to marine waters at certain points in their life, usually during the larval stage. Ricefishes on the other hand do not require marine access, which allowed them to colonize landlocked waters that are more distant to the sea, such as the island’s ancient lakes, where in fact amphidromous species are absent and diadromous species are restricted to eels (*Anguilla* spp.) [25]. This colonization of novel areas ultimately is associated with reduced competition for the available resources, and represents a classic scenario of ecological opportunity, a prime driver of adaptive diversification [4]. Also upstream portions of rivers can be expected to be more species depauperate and therefore less competitive in comparison to lowland portions of the same river. However, they likely also offer less available ecological niches [35, 94], and might be more challenging for fish more common in slow-flowing waters, such as ricefishes, to adapt to.

Given that both the current lineage-poor ichthyofaunal composition of Sulawesi and the phenotypic disparity of its endemic ricefishes match a scenario of ecological opportunity, we conclude it represents a main driver of phenotypic diversification in Sulawesi ricefishes. Nonetheless, Sulawesi is also known for its complicated geological history, reflected in its current geological complexity, e.g. with large highland areas separating island parts and associated drainage basins from each other [112, 116, 117]. This isolation can be expected to have influenced today’s species diversity of Sulawesi ricefishes through long-term spatial isolation. Indeed, substantial genetic distances are reported from several riverine species or species groups in different river systems, including *O. celebensis* [12, 26, 30] and species of the *O. woworae* group [12, 26, 31, 45, 68]. However, while these species are phenotypically highly similar, they often show larger genetic distances than phenotypically far more differentiated lacustrine ricefish species. Still, this discrepancy can readily be explained by the greater ecological opportunity that ricefishes where likely exposed to in lakes. Finally, in addition to the influence of ecological opportunity and geology, there are also signals of ancient hybridization between Sulawesi ricefish lineages (e.g [27]), which possibly further promoted phenotypic variation through gene flow-mediated increases in genomic variation.

## Conclusions

Our results provide the first quantitative assessment of shape diversity in the ricefish family, and particularly the radiation of Sulawesi ricefishes. We demonstrate that the Sulawesi ricefish lineage is phenotypically highly diverse, with exceeding disparity in terms of shape and size compared to related lineages from outside the island. Diversification includes the occupation of previously unoccupied morphospace regions and evolution of extreme traits, suggesting the evolution of novel phenotypes was at least in part driven by ecological opportunity. In addition to shape, also size increased disproportionally in the Sulawesi ricefish lineage. However, miniaturization is exclusively seen in several *Oryzias* species from mainland Asia, where it evolved repeatedly, but absent in Sulawesi ricefishes, likely due to a general absence of many competing or predating lineages that are present in the oriental fauna.

We find indirect evidence through morphospace occupation and disparity, reduced phylogenetic signal and increased rates of shape evolution that adaptive processes related to ecological opportunity in lacustrine environments have greatly influenced the observed phenotype diversity in Sulawesi. Lakes gave rise to more derived morphological shapes, while riverine ricefishes are more confined, often showing greater similarity to taxa from outside Sulawesi. In particular, habitat specialization, and to a lesser extent also diet specialization, appear to be prominent drivers of body and head shape evolution. The ichthyofaunal composition of lakes with sympatric ricefish species suggests that predation and competition with other lineage radiations are possible factors for constrained diversification into sympatric species in spite of ecologically diverse habitats, but apparently were insufficient to prevent diversification entirely at least in one case (Lake Towuti).

Apart from niche adaptation, long-term spatial isolation is another plausible driver of species diversity of Sulawesi ricefishes, reflected in very localized endemism to single lakes or river systems in most species. However, this only provides insufficient explanation for the increased phenotype disparity, contrasted by low phylogenetic signal of phenotype disparity. While it appears most likely that Sulawesi was colonized through marine water by a euryhaline ancestral species, reconstruction of the evolutionary history after colonization is exacerbated by the island’s intricate but insufficiently studied geological history and past introgressive hybridization events. Ongoing species descriptions indicate that knowledge of the taxonomic diversity of ricefishes is still incomplete, stressing the need for more explorative sampling. Together with ecological data, this will contribute to a more detailed and veritable reconstruction of ricefish evolution.

## Electronic supplementary material

Below is the link to the electronic supplementary material.

Supplementary Material 1: Supplementary Tables 1, 3–4, and supplementary Figs. 1–11

Supplementary Material 2: Suppl_Tab_2_Specimen_List.csv: Supplementary Table 2: Spreadsheet listing all museum material that was used for Geometric Morphometric analyses

## Data Availability

The X-Ray scans generated and analyzed in this study are deposited in Morphobank Project 5365 under http://morphobank.org/permalink/?P5365.
