# Supplementary material for: Increased phenotypic diversity as a consequence of ecological opportunity in the island radiation of Sulawesi ricefishes (Teleostei: Adrianichthyidae)
Source: BMC Ecol Evol. 2025 Mar 12;25:19. doi: 10.1186/s12862-025-02355-1 (PMC11900528; doi:10.1186/s12862-025-02355-1)
Supplement: Supplementary file 1 — Supplementary Material 1: Supplementary Tables 1, 3–4, and supplementary Figs. 1–11 [file 12862_2025_2355_MOESM1_ESM.docx]

**Supplementary Figures and Tables**

**Table of contents**

Tab. S1: Taxon samping **2**

Fig. S1: Illustration and description of landmark positions **2**

Fig. S2: Summary of BUSCO assessment results **4**

Fig. S3: Labelled phylomorphospaces for PCs 1 – 3 of body shape species means **5**

Fig. S4: Labelled phylomorphospaces for PCs 1 and 2 of head shape species means **6**

Fig. S5: Labelled phylomorphospace for species means of standard length vs. regression scores **6**

Tab. S3: Explanation of acronyms used in Fig. S3-5 and S7-8 **7**

Fig. S6: Shape changes of PCAs on female-specific body and head shape species means **8**

Fig. S7: Labelled phylomorphospaces for PCs 1 – 3 of female-specific body shape species means **9**

Fig. S8: Labelled phylomorphospaces for PCs 1 – 2 of female-specific head shape species means **10**

Tab. S4 Partial disparity for female-specific head shape species means **11**

Tab. S5 Phylogenetic signal for female-specific head shape species means **11**

Tab. S6 Rates of shape evolution for female-specific head shape species means **11**

Fig. S9: Shape changes of PCAs on body and head shape species means for each sex **12**

Fig. S10: Plot for PCs 1 – 3 on body shape species means for each sex**13**

Fig. S11: Plot for PCs 1 and 2 on head shape species means for each sex**14**

For Tab. S2 (list of specimens used), refer to its separate file.

**Tab. S1**: Taxon samping, with information about taxa, sample size, inclusion in analyses, and sequence data used for Geometric Morphometric and Phylogenomic analyses.

| **Taxon** | **Population** | **MH** | **Sample Size**  **(m/f)** | **GM** | **WGS** | **Assembly Accession Number** | **Project Accession Number** | **SRA Accession Number** |
| --- | --- | --- | --- | --- | --- | --- | --- | --- |
| **Sulawesi species** | | | | | | | | |
| *A. oophorus* |  | L | 9/35 | x | x |  | PRJDB10385 | DRR240750 |
| *A. poptae* |  | L | 1/2 | x | x |  | PRJDB10385 | DRR240751 |
| *O. asinua** |  | R | 0/1 |  | x |  | PRJDB10385 | DRR240752 |
| *O. celebensis* | Asanae | R |  |  | x |  | PRJDB10385 | DRR240753 |
| *O. celebensis* | Malino / Rumbia | R | 2/5 | x | x |  | PRJDB10385 | DRR240754 |
| *O. celebensis* | Ujung Pandang /  Maros | R | 3/3 | x | x |  | PRJDB10371 | DRR240140 |
| *O. doping-dopingensis* |  | R | 5/5 | x | x |  | PRJDB10385 | DRR240755 |
| *O. eversi* |  | R | 21/13 | x | x |  | PRJDB10385 | DRR240756 |
| *O. hadiatyae* |  | L | 6/9 | x | x |  | PRJDB10385 | DRR240757 |
| *O. loxolepis* |  | L | 6/8 | x | x |  | PRJDB10385 | DRR240759 |
| *O.* cf. *marmoratus* | Lantoa | L |  |  | x |  | PRJDB10385 | DRR240760 |
| *O.* cf. *marmoratus* | Mahalona | L |  |  | x |  | PRJDB10385 | DRR240761 |
| *O. marmoratus* | Towuti | L | 1/2 | x | x |  | PRJDB10385 | DRR240758 |
| *O. matanensis* |  | L | 11/8 | x | x |  | PRJDB10385 | DRR240762 |
| *O. nebulosus* |  | L | 37/19 | x | x |  | PRJDB10385 | DRR240763 |
| *O. nigrimas* |  | L | 24/27 | x | x |  | PRJDB10385 | DRR240764 |
| *O. orthognathus* |  | L | 19/19 | x | x |  | PRJDB10385 | DRR240765 |
| *O. profundicola* |  | L | 8/6 | x | x |  | PRJDB10385 | DRR240766 |
| *O. sarasinorum* |  | L | 5/6 | x | x |  | PRJDB10385 | DRR240767 |
| *O. soerotoi* |  | L | 1/4 | x | x |  | PRJDB10385 | DRR240768 |
| *O. wolasi* | Anduna | R | 11/11 | x | x |  | PRJDB10385 | DRR240769 |
| *O. moramoensis* | Moramo Pool | R | 16/12 | x | x |  | PRJDB10385 | DRR240770 |
| *O. moramoensis* | Moramo River | R |  |  | x |  | PRJDB10385 | DRR240771 |
| *O. woworae* | Fotuno | R | 3/11 | x | x |  | PRJDB10385 | DRR240772 |
| *O. woworae* | Balano | R |  |  | x |  | PRJDB10385 | DRR240773 |
| *O. woworae* | Laweau | R |  |  | x |  | PRJDB10385 | DRR240774 |
| *O. woworae* | Motobano | R |  |  | x |  | PRJDB10385 | DRR240775 |
|  |  |  |  |  |  |  |  |  |
| **Non-Sulawesi species** | | | | | | | | |
| *O. curvinotus* |  | - | 16/10 | x | x |  | PRJDB9918 | DRR228808 |
| *O. dancena* |  | - | 12/15 | x | x |  | PRJDB9918 (as *O. carnaticus*) | DRR228807 |
| *O. haugiangensis* |  | - | 10/11 | x | x |  | PRJDB9918 | DRR228809 |
| *O. hubbsi* |  | - |  |  | x |  | PRJDB9918 | DRR228810 |
| *O. javanicus* |  | - | 12/10 | x | x | OJAV_1.1 |  |  |
| *O. latipes* |  | - |  |  | x | ASM223467v1 |  |  |
| *O. luzonensis* |  | - |  |  | x |  | PRJDB9918 | DRR228811 |
| *O. mekongensis* |  | - |  |  | x |  | PRJDB9918 | DRR228812 |
| *O. minutillus* |  | - | 3/9 | x | x |  | PRJDB9918 | DRR228813 |
| *O. pectoralis* |  | - | 13/8 | x | x |  | PRJDB9918 | DRR228814 |
| *O. sinensis* |  | - | 2/4 | x | x | ASM858656v1 |  |  |
| *O. song-khramensis* |  | - |  |  | x |  | PRJDB9918 | DRR228815 |
| *O. uwai** |  | - | 1/0 |  | x |  | PRJDB9918 | DRR228816 |
|  |  |  |  |  |  |  |  |  |
| **Outgroup** | | | | | | | | |
| *Xiphophorus maculatus*** |  | - |  |  | x | GCA_  000241075.1 |  |  |

**MH** = Macrohabitat assignment; L = Lacustrine Sulawesi, R = Riverine Sulawesi, NS = Non-Sulawesi

**GM** = Geometric Morphometric Analyses

**WGS** = Available Whole-Genome Sequence data used for phylogenetic analyses

Genetically divergent populations of the same species originating from different river systems, lakes or other water bodies were treated as distinct OTUs

numbers in red indicate insufficient sample size for inclusion in GM analysis

* species excluded from GM analysis due to insufficient sample size

** used as outgroup


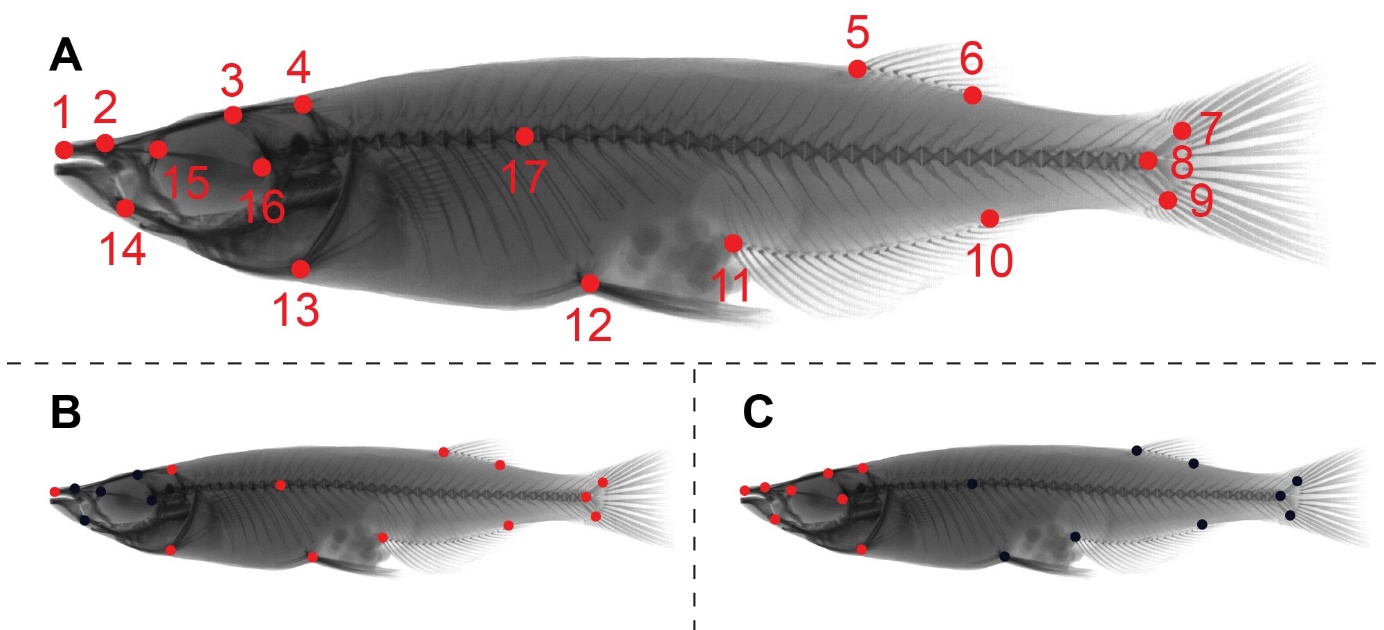


**Fig. S1:** Position of landmarks (LM) used in the geometric morphometric analysis of shape, demonstrated on an X-ray image of a female *Adrianichthys oophorus* (note the abdominal cavity with egg cluster typical for this pelvic-brooding species). A – All landmarks; B – landmark partition used for body shape; C – landmark partition used for head shape.

**LM 1** – Tip of premaxilla; **LM 2** – Upper posterior tip of premaxilla; **LM 3** – Anterior connection postorbital – parietal; **LM 4** – Anterior base of post-temporal; **LM 5, 6** – Pterygiophores of anterior and posterior dorsal fin insertion; **LM 7** – Dorsal tip of epaxial hypural plate; **LM 8** – Connection preural centrum 2 – urostyle; **LM 9** – Ventral tip of hypaxial plate; **LM 10, 11** – Pterygiophores of posterior and anterior anal fin insertion; **LM 12** – Scapula of pelvic fin girdle; **LM 13** – Overlap of cleithrum and base of first branchiostegal; **LM 14** – articular-quadratum joint; **LM 15** – Overlap of parasphenoid and prefrontal; **LM 16** – Posterior cavity of parasphenoid; **LM 17** – narrowest point of vertebrae 9


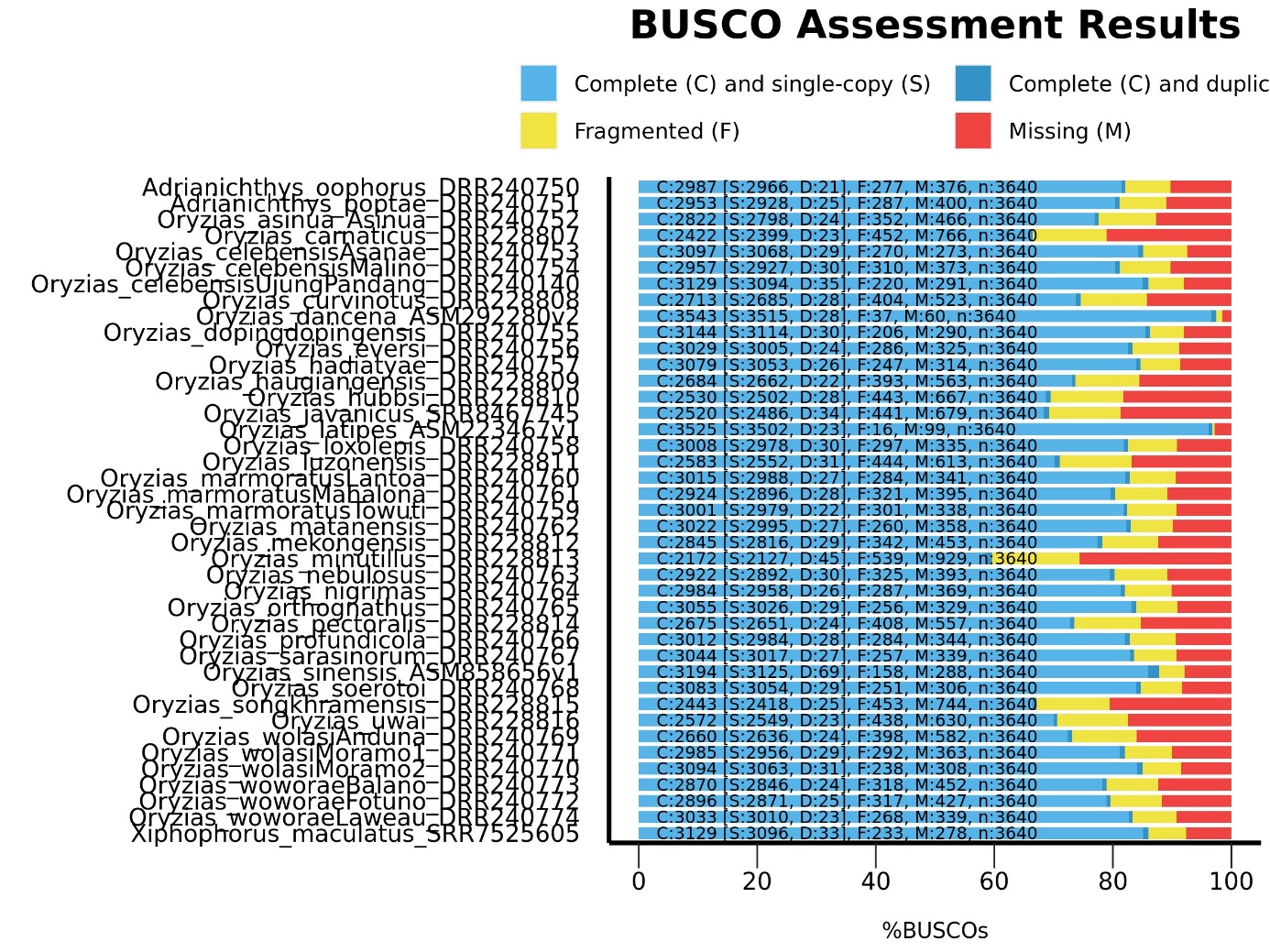


**Fig. S2**: Summary of BUSCO assessment results for the whole genomes reads used in our phylogeny based on the BUSCO set for Actinopterygii. For accession numbers, refer to Tab. S1.

**Labelled Phylomorphospaces**


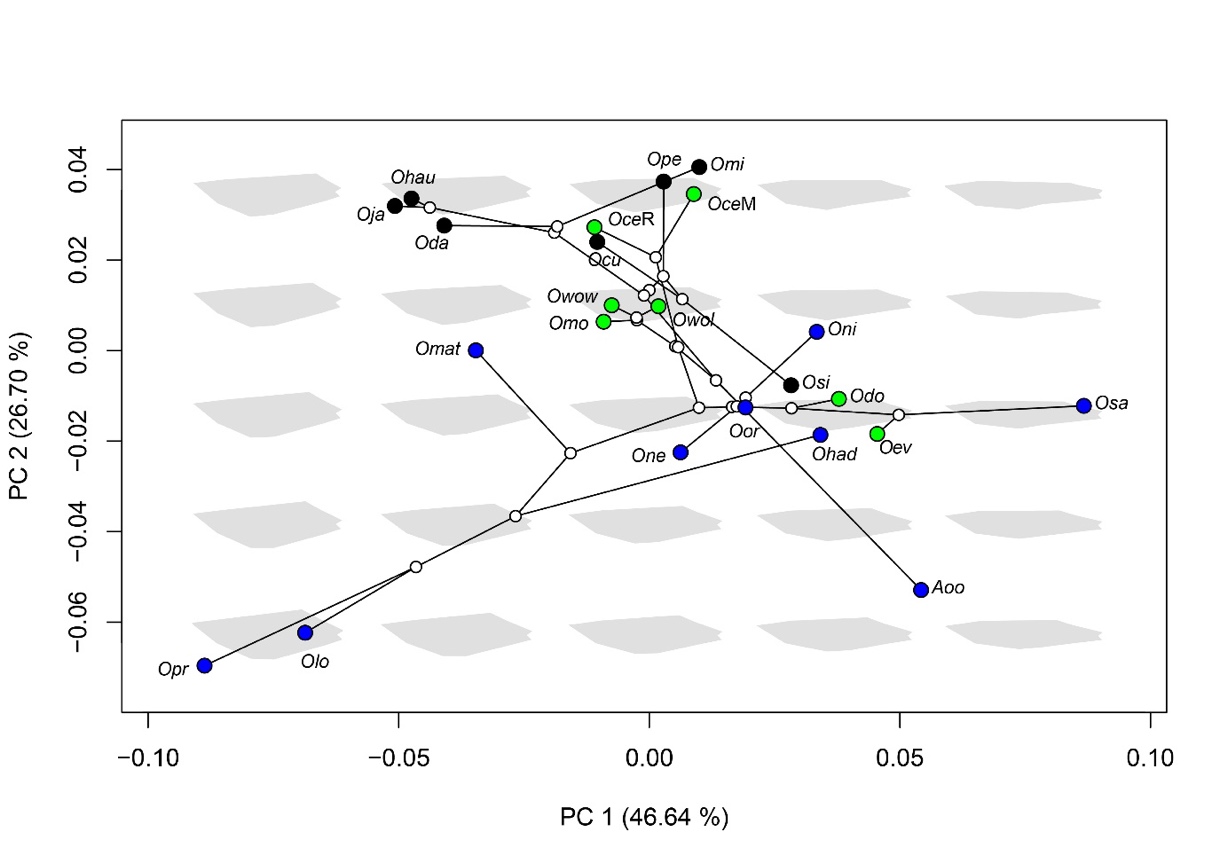


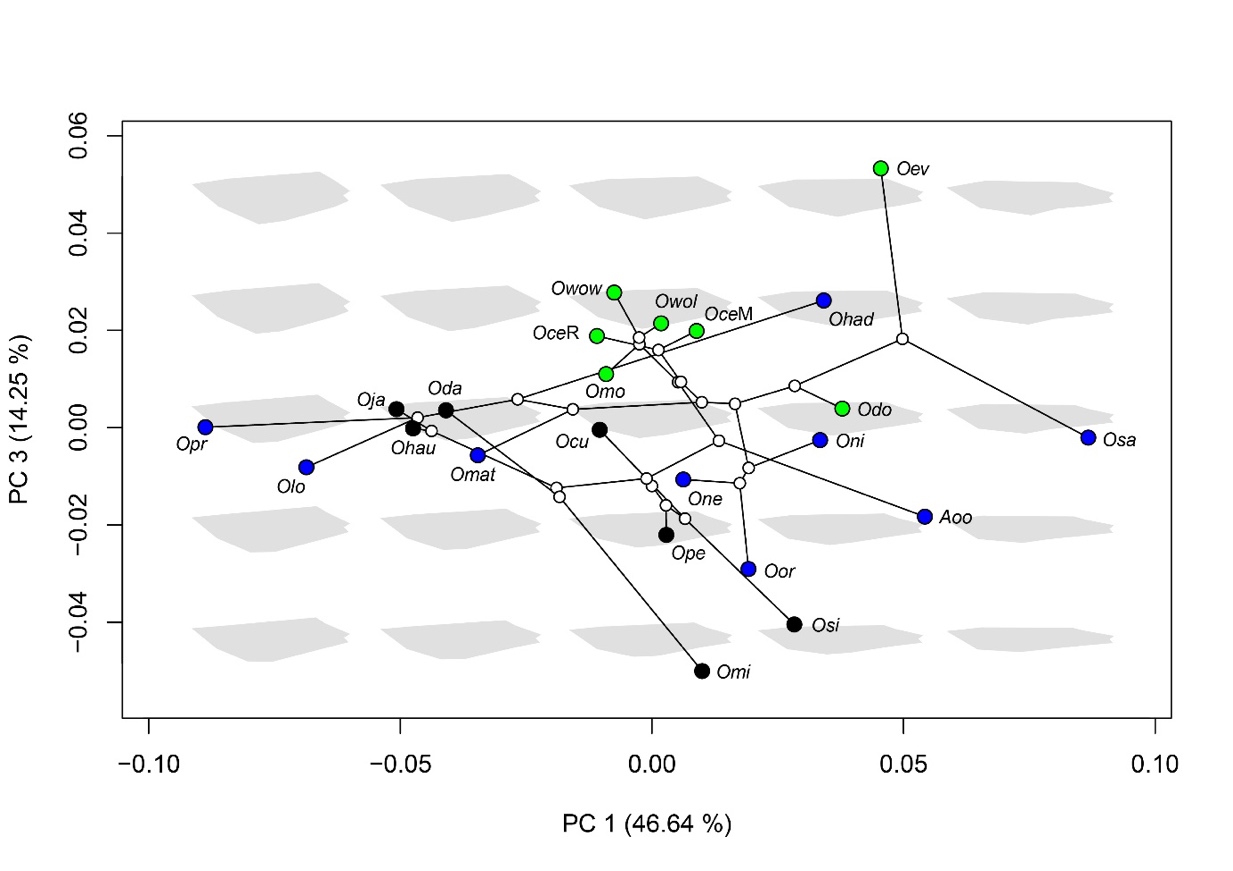


**Fig. S3**: Labelled phylomorphospaces for PCs 1 – 3 of body shape species means. The phylogeny recovered using concatenated BUSCO genes is projected into the morphospace (see Sidlauskas 2008). Taxonomic entities are color coded by macrohabitat group; blue = lacustrine; green = riverine; black = non-Sulawesi. Internal nodes of the phylogeny are colored white and provide estimates for ancestral states. Backtransform morphospaces of the respective PC morphospaces are plotted as grey shapes into the background. These are based on convex hulls of landmark constellations, illustrate shape changes along the PC axes and represent body shape states at the plotting coordinates. For abbreviations of species names see table at the end of this document.


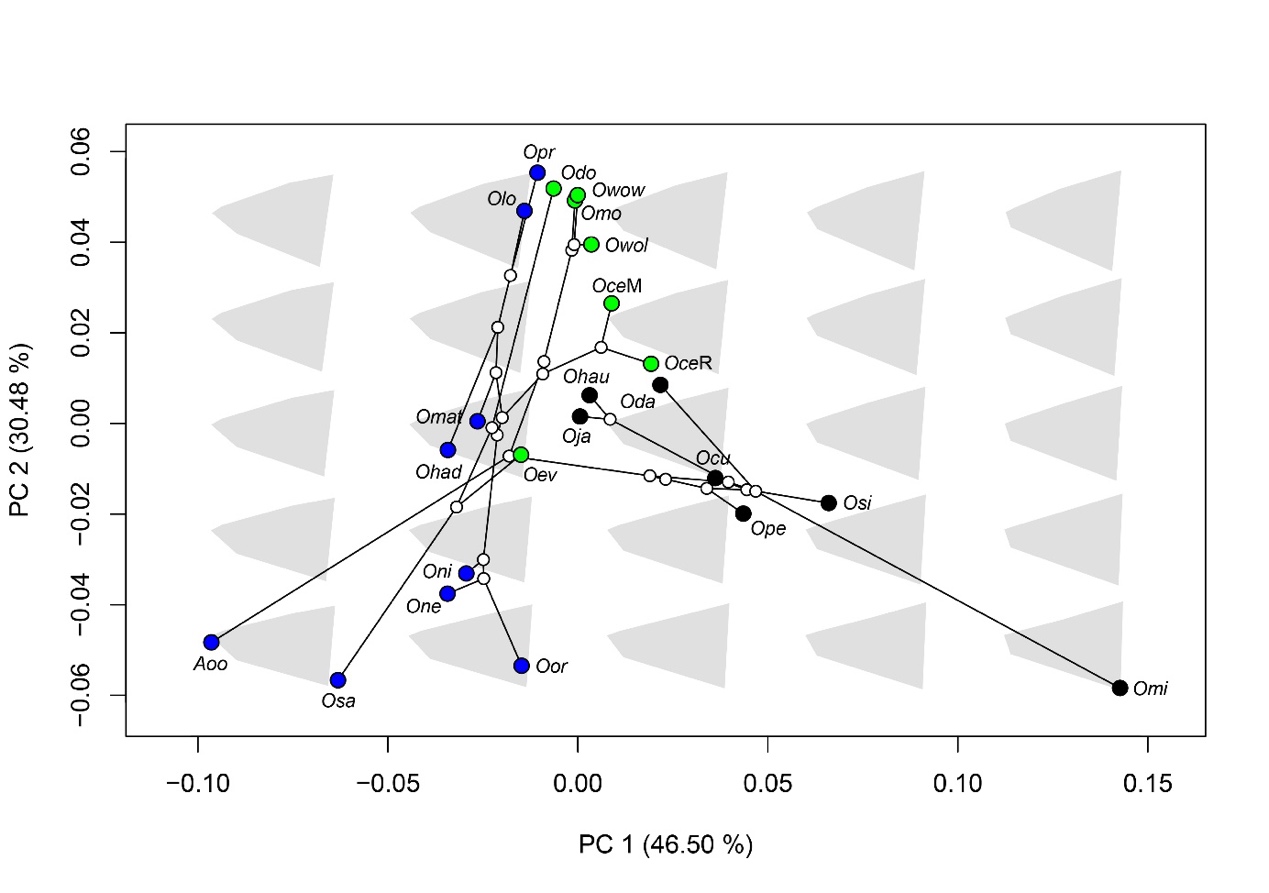


**Fig. S4**: Labelled phylomorphospace for PC 1 and 2 of head shape species means. Colors and shapes analogous to Suppl. Fig. S3.


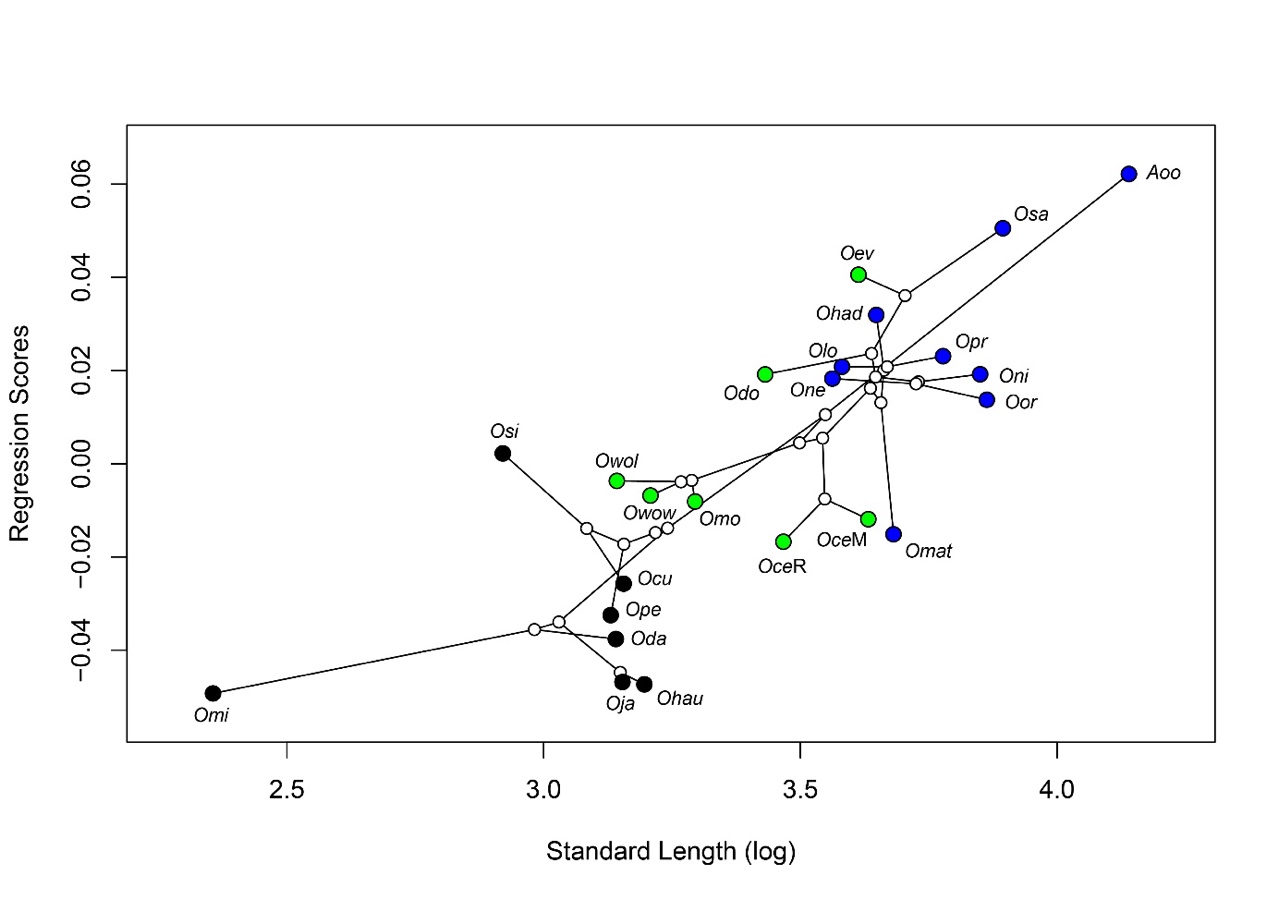


**Fig. S5**: Labelled phylomorphospace with log-transformed standard length on x-axis, and regression scores of Procrustes coordinates of body shape species means against standard length (a measure of allometry) on y-axis. Colors and shapes analogous to Suppl. Fig. S3.

**Tab. S3: Explanation of acronyms used in Fig. S2-4 and S6-7**

| **Taxon** | **Population** | **Abbreviation** | **Comment** |
| --- | --- | --- | --- |
|  |  |  |  |
| *A. oophorus* |  | *Aoo* |  |
| *A. poptae* |  | *Apo* | Excluded from analysis of general species means, only females available |
| *O. celebensis* | Rumbia | *Oce*R |  |
| *O. celebensis* | Maros | *Oce*M |  |
| *O. curvinotus* |  | *Ocu* |  |
| *O. dancena* |  | *Oda* |  |
| *O. dopingdopingensis* |  | *Opo* |  |
| *O. eversi* |  | *Oev* |  |
| *O. hadiatyae* |  | *Ohad* |  |
| *O. haugiangensis* |  | *Ohau* |  |
| *O. javanicus* |  | *Oja* |  |
| *O. loxolepis* |  | *Olo* |  |
| *O. matanensis* |  | *Omat* |  |
| *O. marmoratus* | Lake Towuti | *Omar* | Excluded from analysis of general species means, only females available |
| *O. minutillus* |  | *Omi* |  |
| *O. moramoensis* |  | *Omo* |  |
| *O. nebulosus* |  | *One* |  |
| *O. nigrimas* |  | *Oni* |  |
| *O. orthognathus* |  | *Oor* |  |
| *O. pectoralis* |  | *Ope* |  |
| *O. profundicola* |  | *Opr* |  |
| *O. sarasinorum* |  | *Osa* |  |
| *O. sinensis* |  | *Osi* |  |
| *O. soerotoi* |  | *Oso* | Excluded from analysis of general species means, only females available |
| *O. wolasi* |  | *Owol* |  |
| *O. woworae* |  | *Owow* |  |

**Results of Female-Extended Dataset (dataset ‘DS-FE’)**


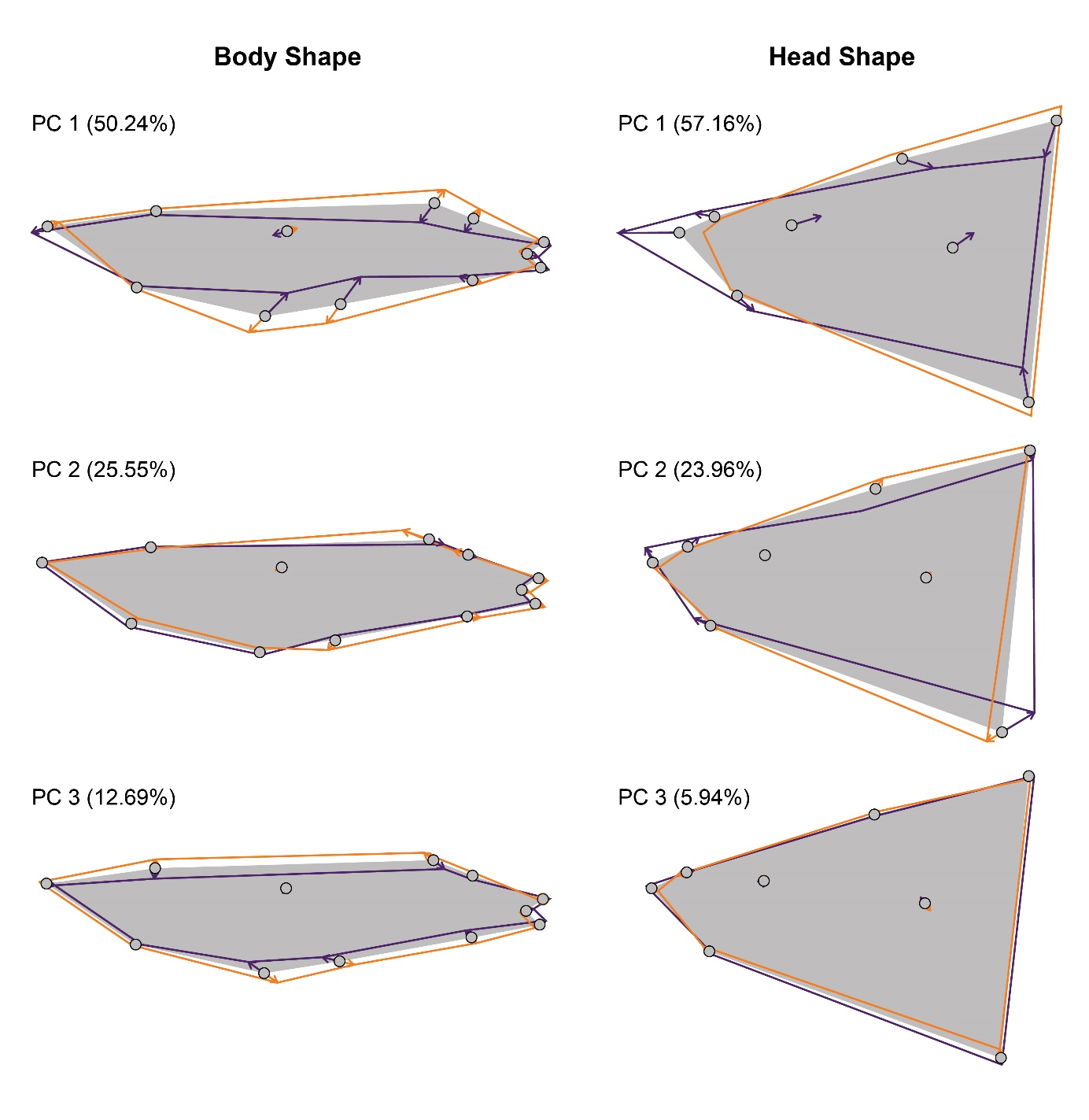


**Fig. S6:** Figures above illustrate the shape trajectories recovered as explaining the greatest variance in the first, second and third principal component of the PCAs on body and head shape variation based on mean shapes of females of each species. These include species that were omitted in the main analysis based on species means (derived from sex-specific mean shapes of species groups) due to absence of male specimens in our sample – namely *Adrianichthys poptae*, *Oryzias marmoratus*, and *O. soerotoi*. Mean shapes of the PC are illustrated as a grey shape, whereas minimum and maximum states along the trajectories are given as orange and purple outlines, respectively.


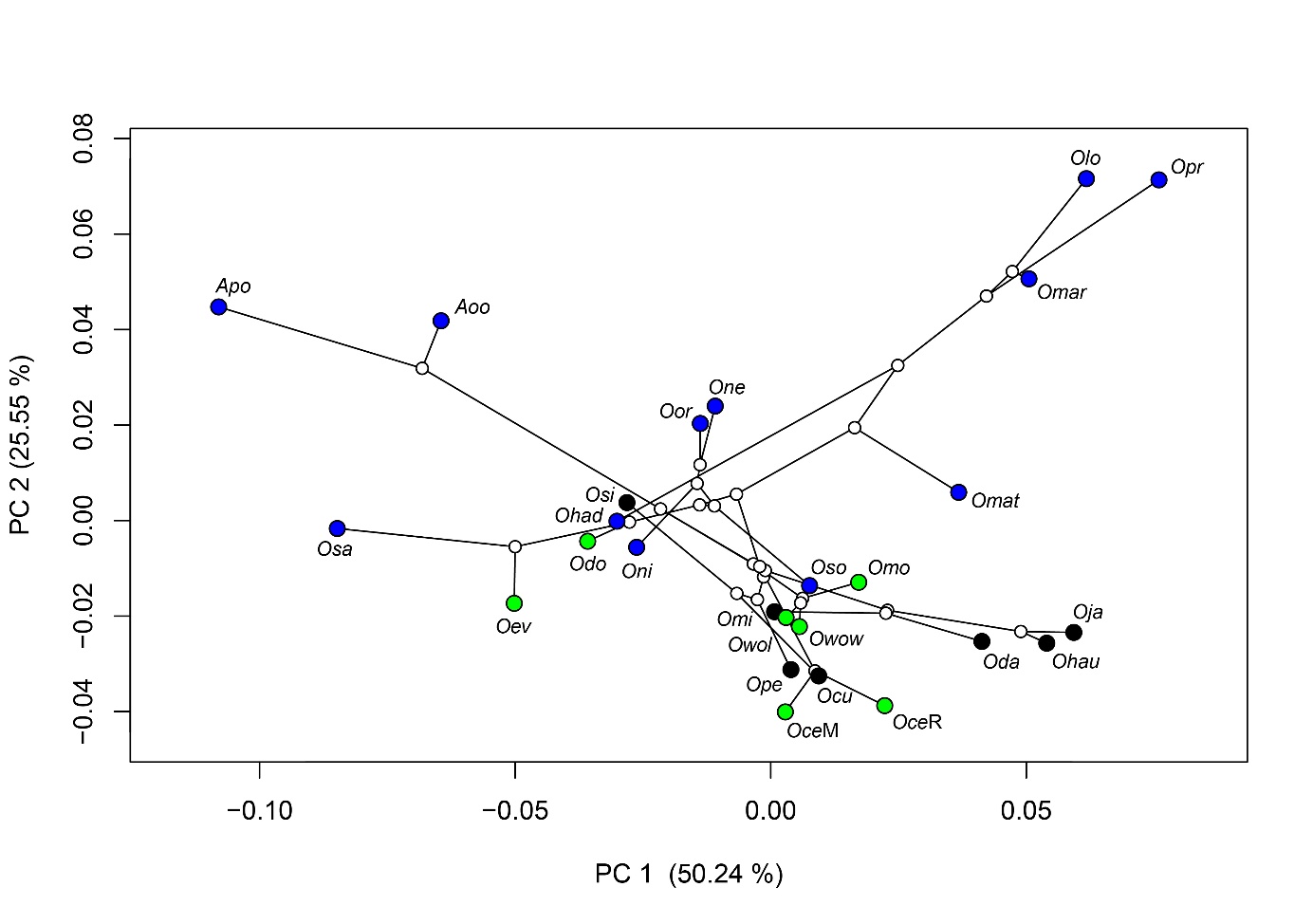


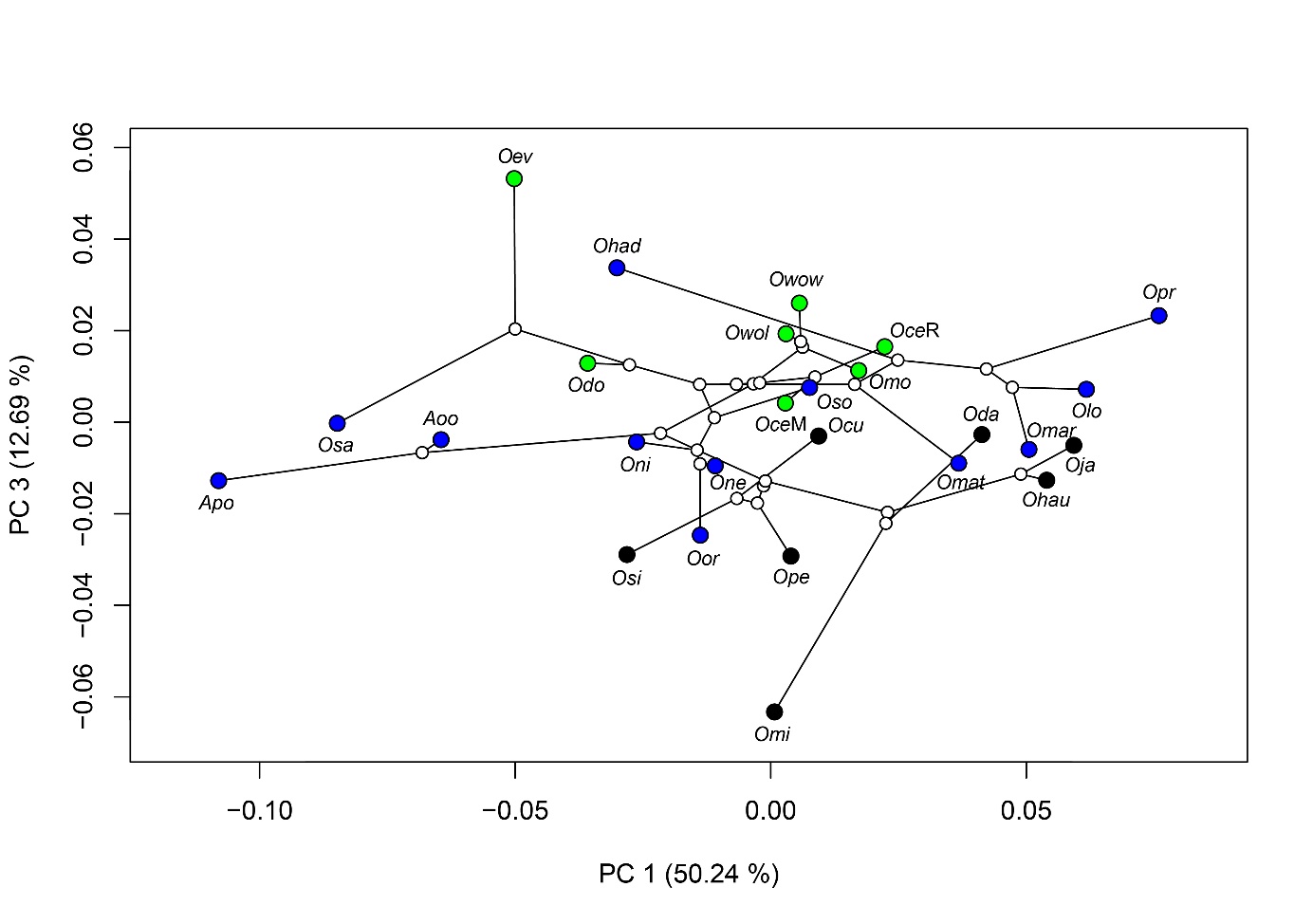


**Fig. S7**: Labelled Phylomorphospaces for female-specific species means of body shape. The phylogeny recovered using concatenated BUSCO genes is projected into the morphospace (see Sidlauskas 2008). Taxonomic entities are color coded by macrohabitat group; blue = lacustrine; green = riverine; black = non-Sulawesi. Internal nodes of the phylogeny are colored white and provide estimates for ancestral states. For abbreviations of species names see Suppl. Tab. S3.


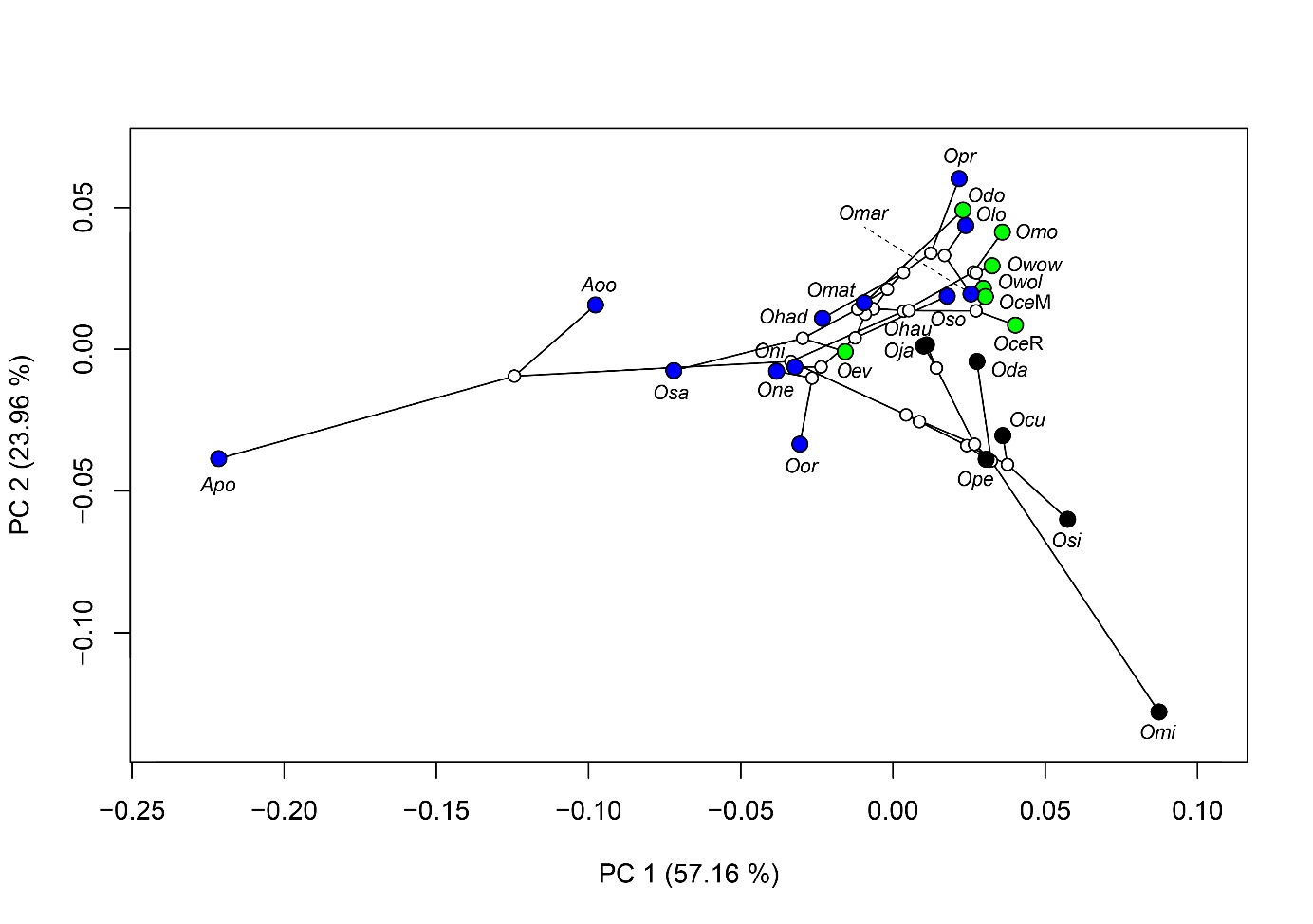


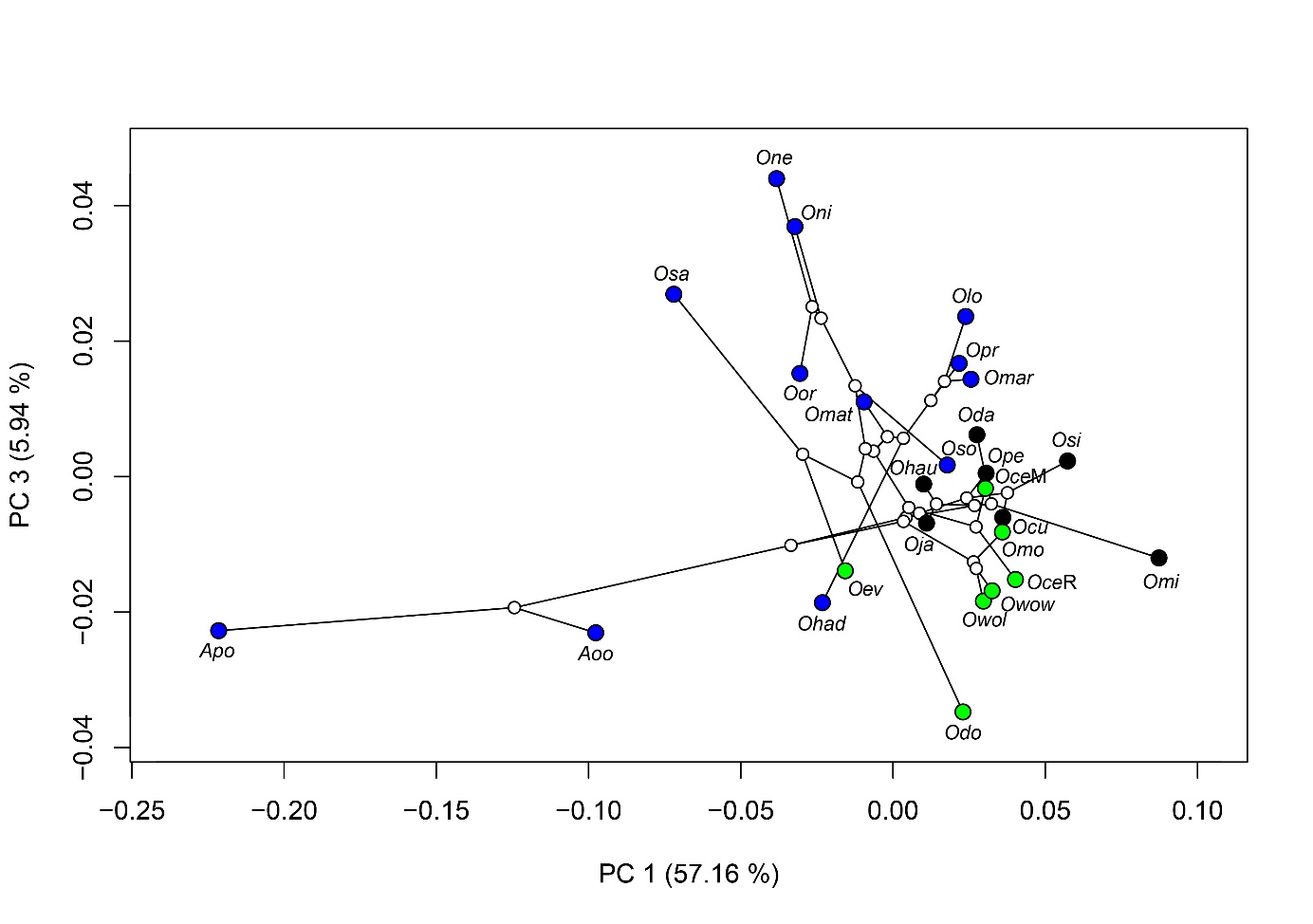


**Fig. S8**: Labelled Phylomorphospaces for female-specific species means of head shape. The phylogeny recovered using concatenated BUSCO genes is projected into the morphospace (see Sidlauskas 2008). Taxonomic entities are color coded by macrohabitat group; blue = lacustrine; green = riverine; black = non-Sulawesi. Internal nodes of the phylogeny are colored white and provide estimates for ancestral states. For abbreviations of species names see Suppl. Tab. S3.

**Tab. S4** Partial disparity (Foote 1993) for body and head shape of female-specific species means. Upper triangle shows pairwise differences between group variances, lower triangle shows *p* values.

|  |  | **Riverine** | **Lacustrine** | **Non-Sulawesi** |
| --- | --- | --- | --- | --- |
| **Body shape** | Procrustes variance | 0.692 x 10^-3^  (16.55%) | 2.614 x 10^-3^  (63.53%) | 0.875 x 10^-3^  (20.93%) |
|  | **Riverine** | **-** | 1.922 x 10^-3^ | 0.183 x 10^-3^ |
|  | **Lacustrine** | *0.027* | **-** | 1.739 x 10^-3^ |
|  | **Non-Sulawesi** | *0.758* | *0.062* | **-** |
| **Head shape** | Procrustes variance 3 | 0.712 x 10^-3^  (10.96%) | 4.013 x 10^-3^  (61.79%) | 1.770 x 10^-3^  (27.25%) |
|  | **Riverine** | **-** | 3.301 x 10^-3^ | 1.058 x 10^-3^ |
|  | **Lacustrine** | *0.178* | **-** | 2.243 x 10^-3^ |
|  | **Non-Sulawesi** | *0.571* | *0. 405* | **-** |

**Tab. S5:** Phylogenetic Signal k and respective p value for body and head shape of female-specific species means.

|  | **Phylogenetic Signal *k*** | ***p* value** |
| --- | --- | --- |
| **Body Shape** | 0.375 | 0.001 |
| **Head Shape** | 0.521 | 0.001 |

**Tab. S6**: Evolutionary rates for body and head shape of female-specific species means.

|  | **Group** | | | ***p* value** |
| --- | --- | --- | --- | --- |
|  | Sulawesi lacustrine | Sulawesi  riverine | Non-Sulawesi |  |
| **Body Shape** | 5.30E-05 | 1.84E-05 | 5.87E-06 | 1.00E-04 |
| **Head Shape** | 8.84E-05 | 2.89E-05 | 2.09E-05 | 1.00E-04 |

**Results of Dataset for assessment of sexual dimorphism (dataset ‘DS-G-MF’)**


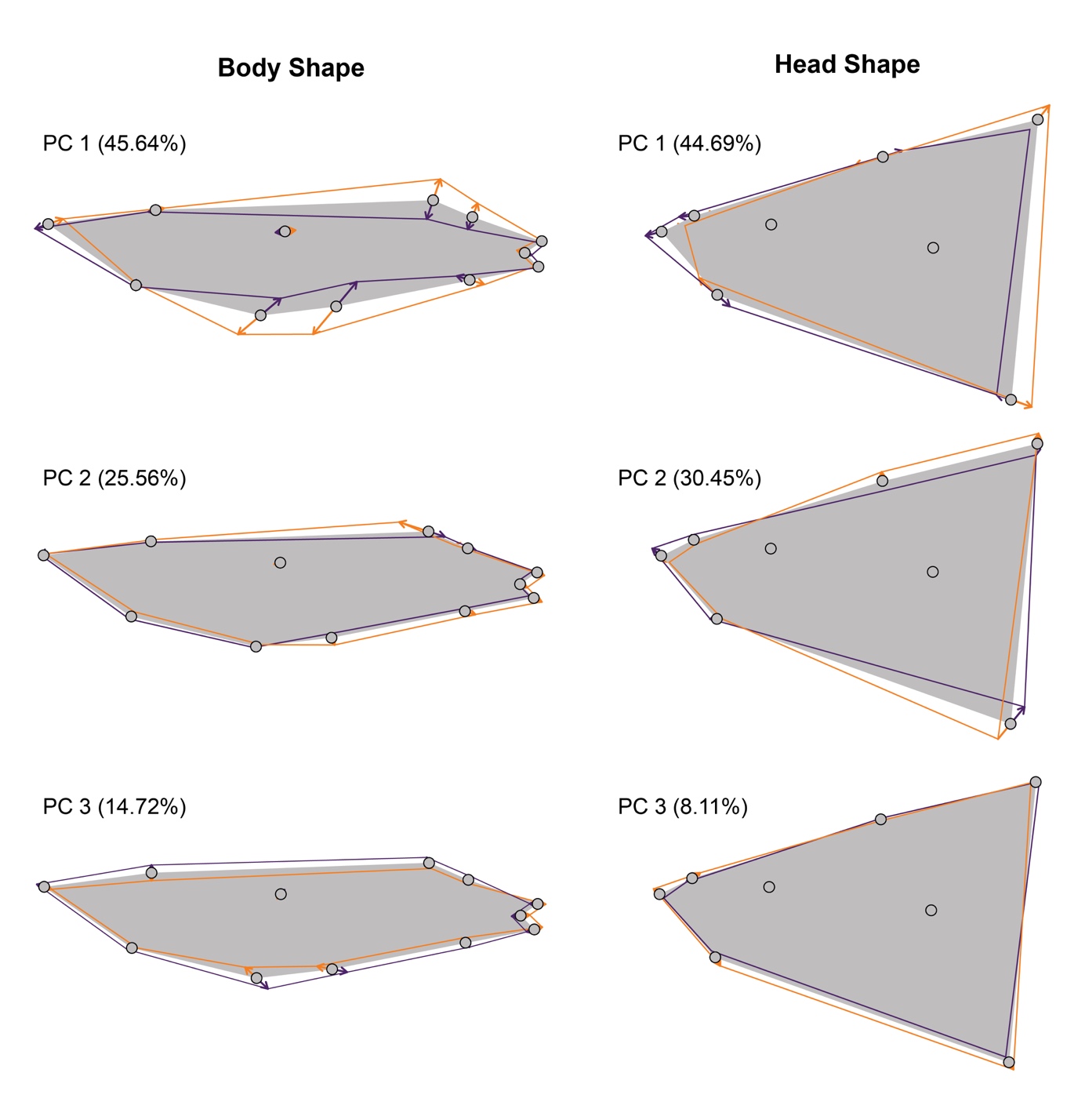


**Fig. S9:** Figures above illustrate the shape trajectories recovered as explaining the greatest variance in the first, second and third principal component of the PCAs on body and head shape variation based on sex-specific species group means, i.e., mean shapes of males and females of each species. Mean shapes of the PC are illustrated as a grey shape, whereas minimum and maximum states along the trajectories are given as orange and purple outlines, respectively.


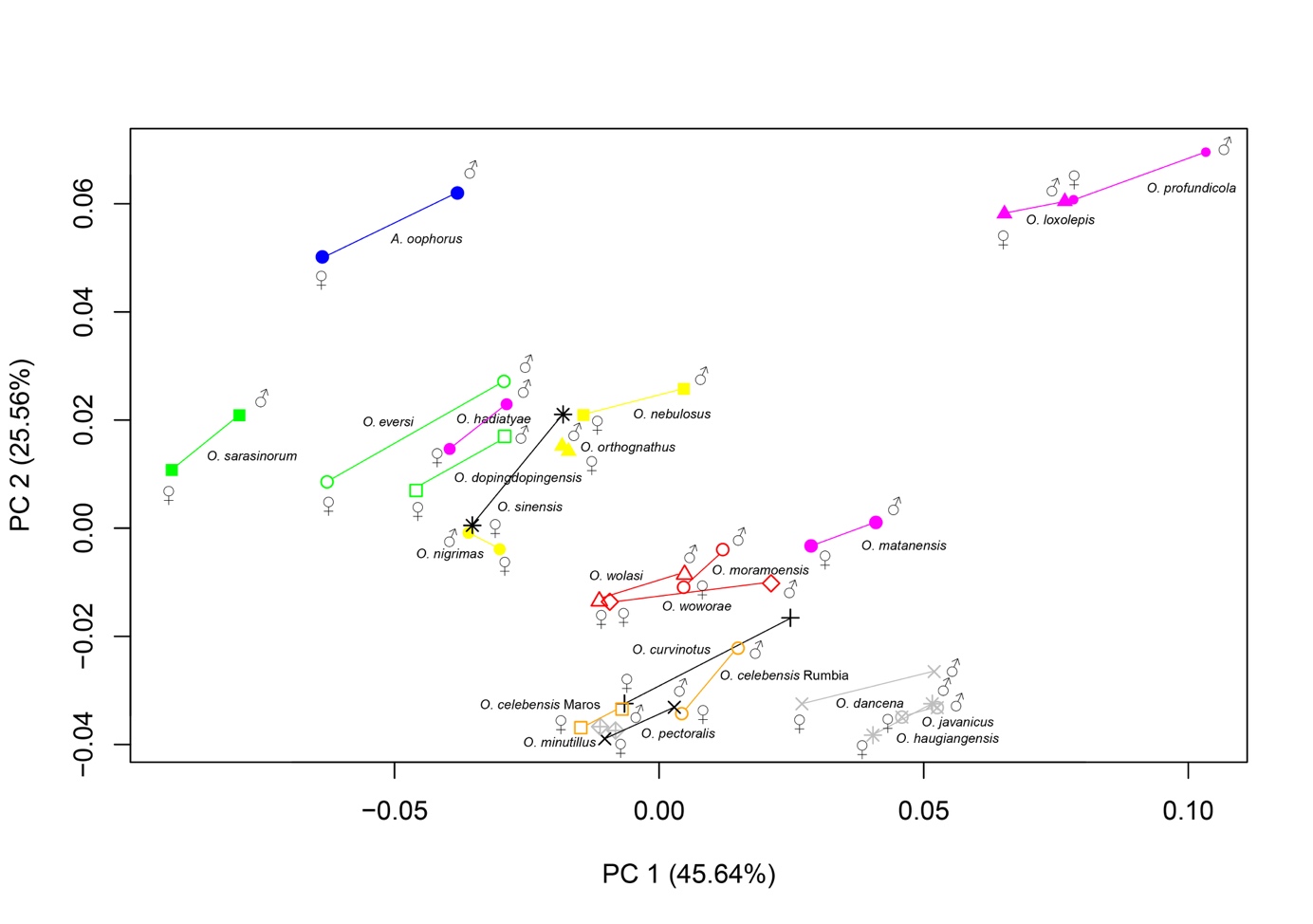


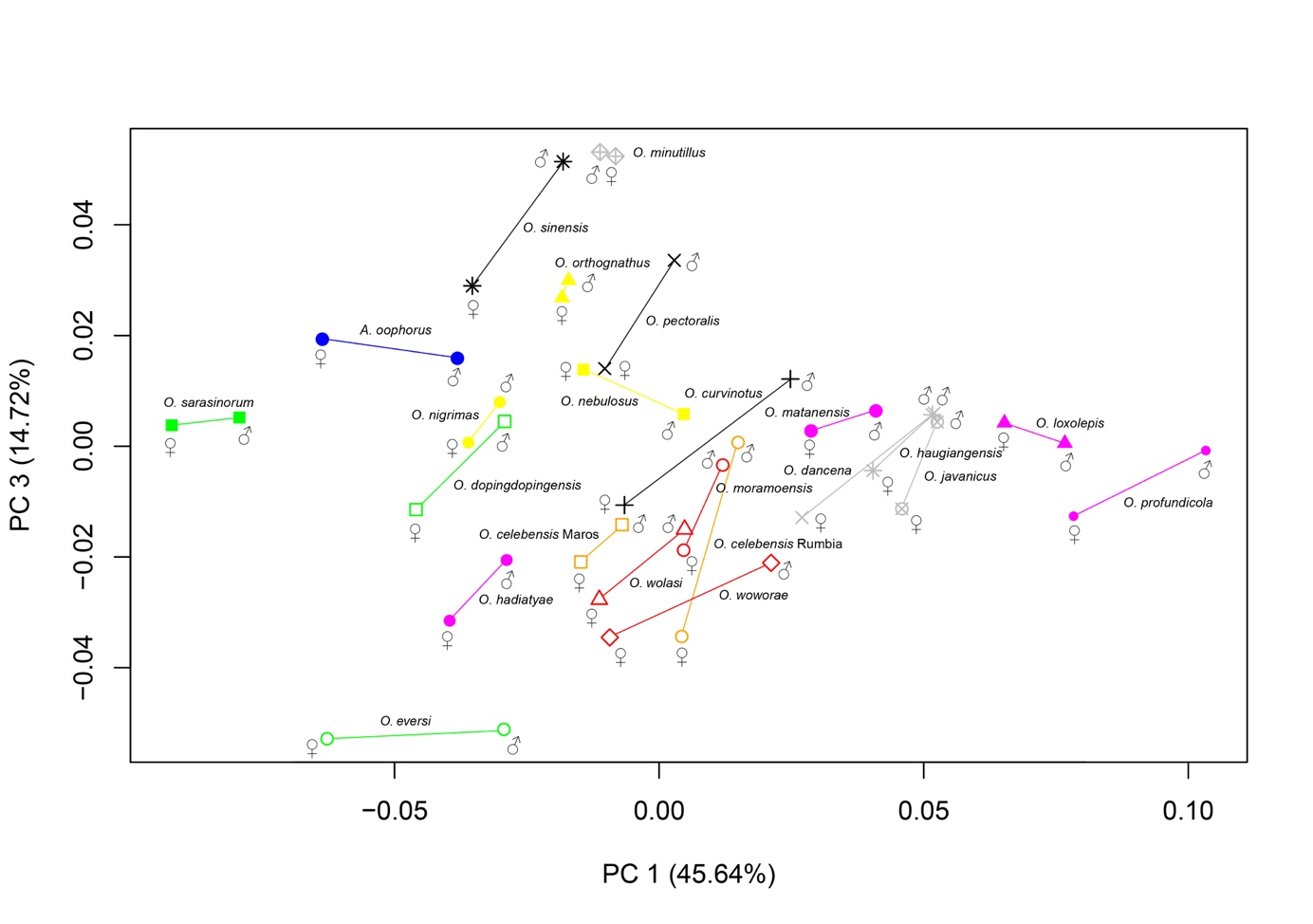


**Fig. S10:** Results of PCA on body shape. Lines connect both sexes of each species, illustrating their distance from each other in morphospace. Sexes are indicated with their respective symbol. Symbol color of each species follows affiliation with major phylogenetic clades as proposed by Mokodongan & Yamahira (2015).


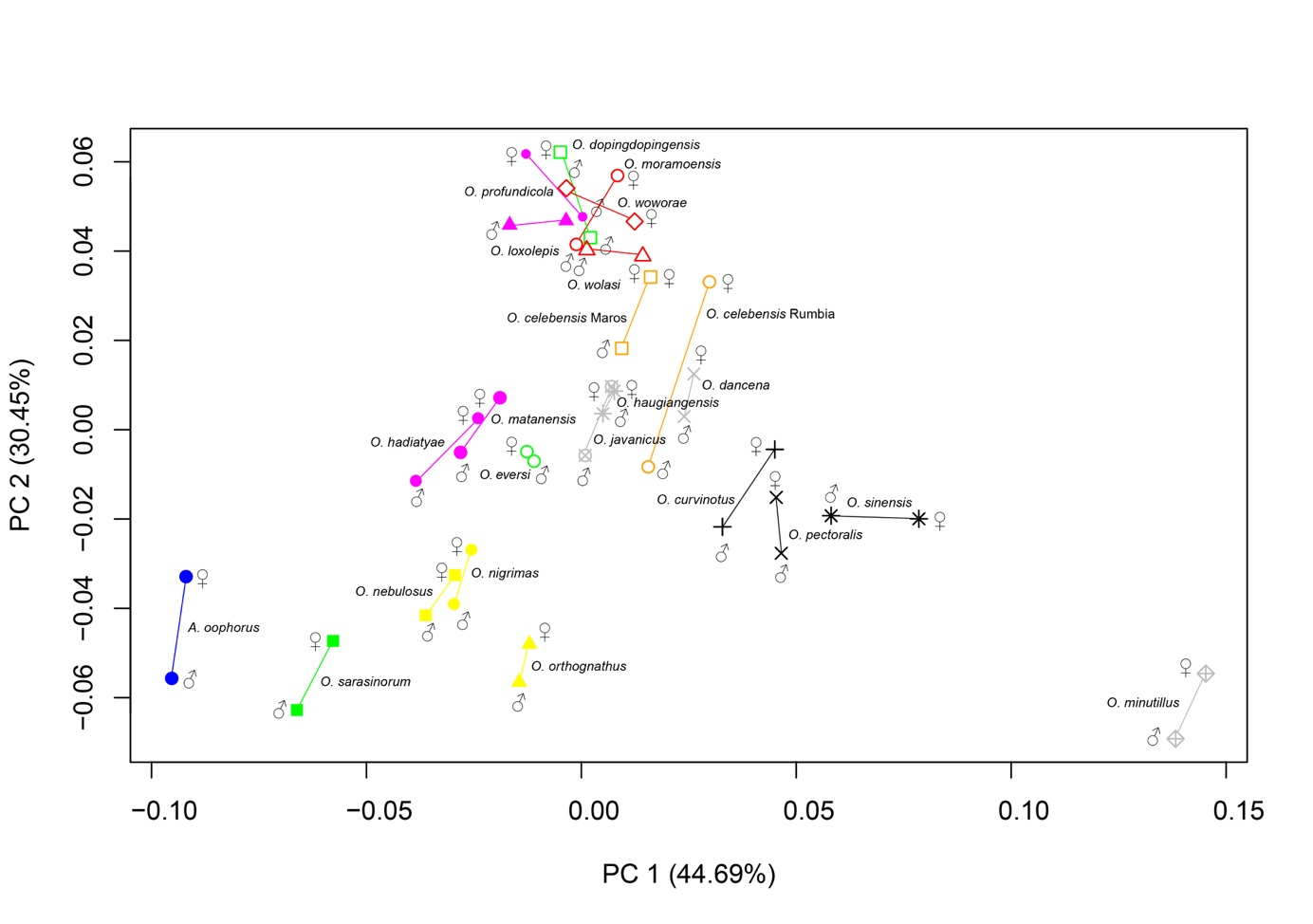


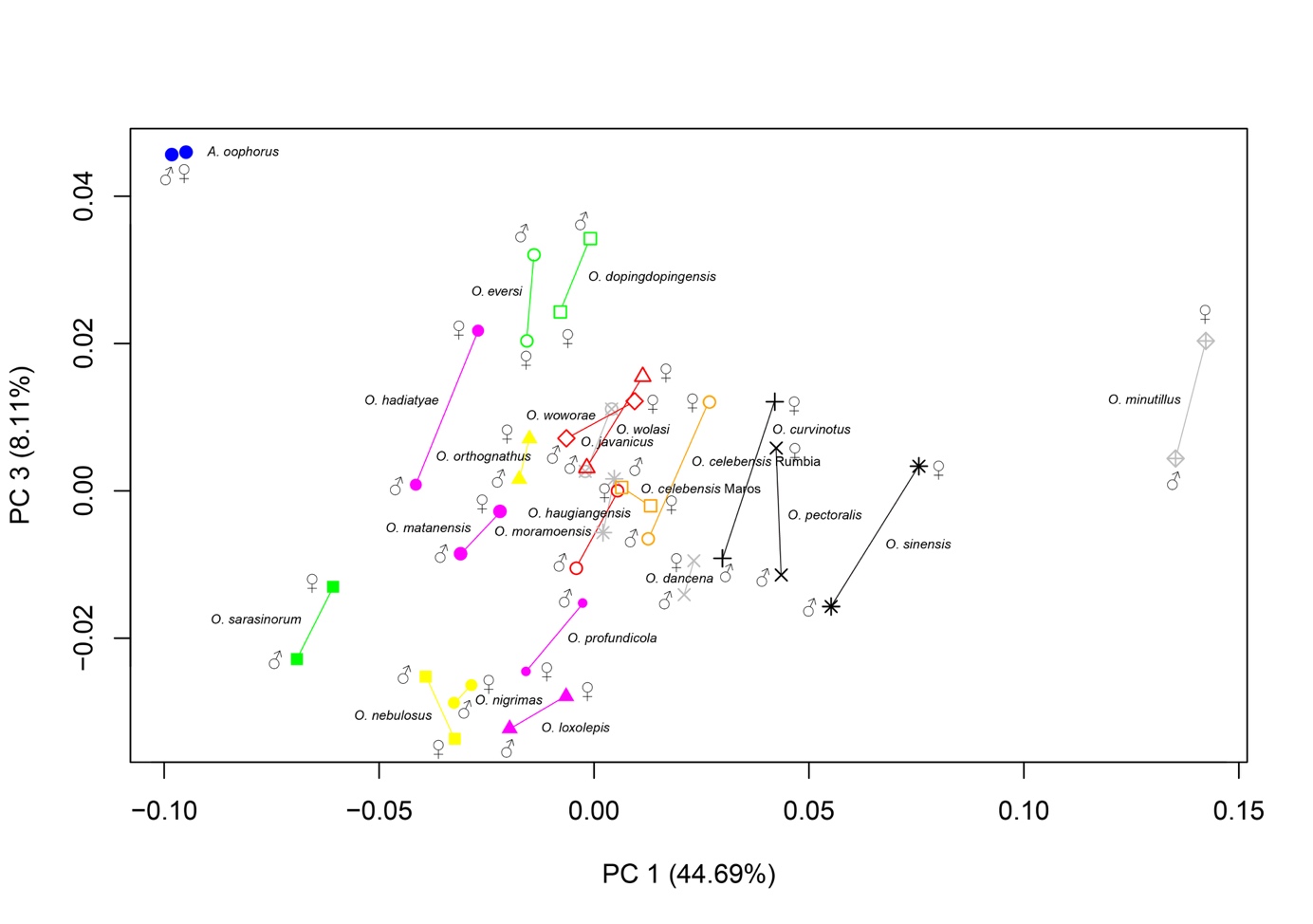


**Fig. S11:** Results of PCA on head shape. Lines connect both sexes of each species, illustrating their distance from each other in morphospace. Sexes are indicated with their respective symbol. Symbol color of each species follows affiliation with major phylogenetic clades as proposed by Mokodongan & Yamahira (2015).
